# Scientific research on food environments in Brazil: a scoping review

**DOI:** 10.1017/S1368980023000836

**Published:** 2023-10

**Authors:** Larissa Loures Mendes, Luana Lara Rocha, Laís Vargas Botelho, Mariana Carvalho de Menezes, Paulo César Pereira de Castro Júnior, Alex Oliveira da Camara, Leticia de Olivera Cardoso, Inês Rugani Ribeiro de Castro, Paula Martins Horta, Milene Cristine Pessoa, Marcela Boro Veiros, Daniela Silva Canella

**Affiliations:** 1 Department of Nutrition, Universidade Federal de Minas Gerais, Belo Horizonte, Minas Gerais 30130-100, Brazil; 2 Department of Preventive and Social Medicine, Universidade Federal de Minas Gerais, Belo Horizonte, Brazil; 3 Escola Nacional de Saúde Pública Sergio Arouca, Fundação Oswaldo Cruz, Rio de Janeiro, Brazil; 4 School of Nutrition, Federal University of Ouro Preto, Ouro Preto, Brazil; 5 Institute of Nutrition Josué de Castro, Federal University of Rio de Janeiro, Rio de Janeiro, Brazil; 6 Department of Social Nutrition, Institute of Nutrition, Rio de Janeiro State University, Rio de Janeiro, Brazil; 7 Department of Nutrition, Federal University of Santa Catarina, Florianopolis, Brazil; 8 Department of Applied Nutrition, Institute of Nutrition, Rio de Janeiro State University, Rio de Janeiro, Brazil

**Keywords:** Food environment, Brazil, Scientific research

## Abstract

**Objective:**

To map the scientific research on food environments in Brazil, based on the following questions: How many studies have addressed food environments?; What study designs and methodological approaches were applied?; What is the geographic scope of the studies?; What scenarios and dimensions of food environments were studied?; Which population groups were studied?; How were food environments conceptualised?; What are the main limitations of the studies?

**Design:**

Scoping review conducted in four databases, from January 2005 to December 2022, using different food environment-related terms to cover the main types and dimensions proposed in the literature. The studies were independently selected by two authors. A narrative synthesis was used to summarise the findings.

**Setting:**

Brazil.

**Participants:**

130 articles.

**Results:**

Scientific research on Brazilian food environments has been increasing. The analytical quantitative approach and the cross-sectional design were the most frequently used. Most articles were published in English. The majority of studies evaluated the community food environment, addressed aspects of the physical dimension, sampled the adult population, had food consumption as an outcome, used primary data, and were carried out in capital cities in the Southeast region. Furthermore, in most articles, no conceptual model was explicitly adopted.

**Conclusions:**

Gaps in literature are related to the need for conducting studies in the Brazilian countryside, the support for the formulation of research questions based on conceptual models, the use of valid and reliable instruments to collect primary data, in addition to the need for a greater number of longitudinal, intervention and qualitative studies.

Food systems can impact the health of populations in different ways, and they have been identified as one of the drivers of the global syndemic of obesity, undernutrition and climate change^(1)^. One of the components of food systems is the food environment, which acts as a mediator between food supply chains and food practices, and it encompasses the availability, affordability, convenience and desirability of food. Thus, food environments influence decisions about food acquisition, preparation and consumption – the latter, in turn, interferes with people’s nutritional status^(2–4)^.

Knowledge and understanding of food environments, including where, what, by whom, when, why, and how food is acquired and consumed, have been considered fundamental aspects to understand the phenomenon of malnutrition in all its forms^(3)^. Previous review studies indicated a wide range of conceptual models, methods and metrics to characterise and measure the different dimensions of food environments^(5–9)^. They also showed that unhealthy food environments are associated with unhealthy food practices and with health outcomes related to overweight and obesity^(10,11)^. Furthermore, they pointed out that different population groups are disproportionately affected, that is, low-income people and ethnic minorities are more exposed to unhealthy environments^(10,11)^. However, research on this topic is most often conducted in high-income contexts; for this reason, food environments must be characterised in different places, such as low- and middle-income countries, and cities of different sizes.

In the scenario of scientific research on food environments, there is controversy about the role of Brazil in this research agenda. Turner et al.^(3)^ stated that research on food environments in middle- and low-income countries, such as Brazil, was still incipient, and they proposed a conceptual model to support and leverage the research in the context of such countries. On the other hand, Pérez-Ferrer et al.^(12)^ highlighted the leading role of Brazil in scientific research on food environments in Latin America, a fact that was also pointed out in the scoping review by Muzenda et al.^(8)^, who identified methods for evaluating food environments in low- and middle-income countries, thereby reinforcing the role of Brazil in studies on this topic. However, in another scoping review, Turner et al.^(7)^ highlighted again both the scarce scientific literature on the subject and the low quality of the studies, and they underscored the reduced number of publications in Brazil.

There is considerable controversy concerning the quality and the number of studies on food environments in Brazil – a fact that has been mentioned in different publications. Moreover, there is a growing interest in the current research scenario on the subject across the country. Therefore, the objective of the present scoping review was to map the scientific research on food environments in Brazil and identify knowledge gaps in order to promote a comprehensive research agenda and contribute to the formulation of public policies.

## Methods

The present scoping review summarised the scientific research on food environments in Brazil, following the checklist and guidelines of the *Preferred Reporting Items for Systematic Reviews and Meta-Analyses* – *Extension for Scoping Reviews* (PRISMA-ScR)^(13)^, aiming to ensure a robust and reproducible process. This review did not have a previously registered protocol.

### Research questions

Considering the objective to map the scientific research on food environments in Brazil, the review had these research questions: (1) How many studies have addressed food environments in Brazil?; (2) Which institutions have conducted most research on this topic in the country?; (3) What study designs and methodological approaches were applied?; (4) What is the geographic scope of the studies?; (5) What scenarios and dimensions of food environments were studied?; (6) Which population groups were studied?; (7) How were food environments conceptualised?; (8) What are the main limitations of studies on food environments in Brazil?

### Search strategy

In February 2023, a systematic search was conducted for articles published and peer-reviewed from 1 January 2005 to 31 December 2022. This period was chosen as a starting point because of the publication of the conceptual model of food environments proposed by Glanz et al.^(14)^.

Considering the still scarce adequacy of descriptors for the food environment theme, according to the *Medical Subject Heading*s – MeSH and the descriptors in health sciences – DeCS, different search terms were used to capture the breadth of the nomenclature adopted in research and the scope of the main dimensions of food environments, according to different conceptual models^(4,14,15)^. The terms food environment or nutrition environment were used in combination with community, organisational, consumer, information, home, school, digital, virtual, perceived, observed, neighbourhood, retail, local, urban, natural, built, formal, informal, university, hospital, workplace, food swamp and food desert.

The search was conducted in four electronic databases: MEDLINE, SciELO, Scopus and Web of Science. The Boolean operator OR was used for terms that defined different types or dimensions of food environments, and they were combined using the AND operator with the name of the country. The descriptors used and the search strategy adopted in each database are described in Supplementary Material S1.

### Eligibility criteria

The present review included articles published online in peer-reviewed journals, between January 2005 and December 2022, that described or evaluated food environments in Brazil or investigated their association with diet, nutritional status, or other health outcomes. To this end, a broad strategy was used, covering quantitative, qualitative and mixed-method studies, as well as comments or review articles or opinions on some aspects of food environments. There were no restrictions on the language of publication. The following were excluded: (a) studies carried out outside Brazil; (b) conference abstracts; (c) articles on topics related to the food systems with a focus on food production, storage, transport, distribution and food security which did not address a direct perspective of food environments.

### Selection of studies

First, all retrieved records were transferred to the software Endnote V.X9 to remove duplicates. Then, two authors (LVB and LLR) selected the studies independently, according to the eligibility criteria. All titles and abstracts were read to check inclusion and exclusion criteria. Then, the full texts of the articles were retrieved and screened. At each selection step, disagreements were resolved by discussion between the authors involved in the screening procedure. Whenever a consensus could not be achieved, a third reviewer (DSC) was asked to evaluate the article in question and offer an opinion.

All articles were published in journals approved by the Directory of Open Access Journals (DOAJ).

### Data extraction and analysis

The data were extracted using a standardised data extraction form, developed by two reviewers (LVB and LLR) in Google Forms. This instrument was previously tested by these evaluators in a random sample of ten articles and refined after being checked by the other authors to ensure that all relevant information had been retrieved. Each reviewer extracted data from half of the articles and had their work carefully checked by the other reviewer. Disagreements were also resolved between the authors in charge of data extraction and, if necessary, a third author (DSC) was involved.

The extracted variables were title, year of publication, journal name, institution affiliation of the first author, country of the institution of the first author, language, study objective, macroregion where the study was conducted, geographic coverage, methodological approach, study design, study population, sampling plan, focus on food environments, data source, explicit adoption of conceptual food environment models and their specification (when applicable), type of food environment, dimension of the food environment, health and nutrition outcome assessed, the instrument used to collect data on food environments and limitations pointed out by the authors concerning food environment data. Concerning the type and the dimension of the food environment, the definition of type was based on Glanz^(14)^ et al and Granheim^(15)^, and the dimension was based on Swinburn et al.^(16)^. Despite not all studies mentioning the adoption of these conceptual models, we proceeded with the classification of each study according to them.

The extracted information was described using absolute and relative frequencies to help the narrative synthesis of the findings. As expected in a scoping review, the quality of the studies was not evaluated^(13)^.

## Results

**Fig. 1:**
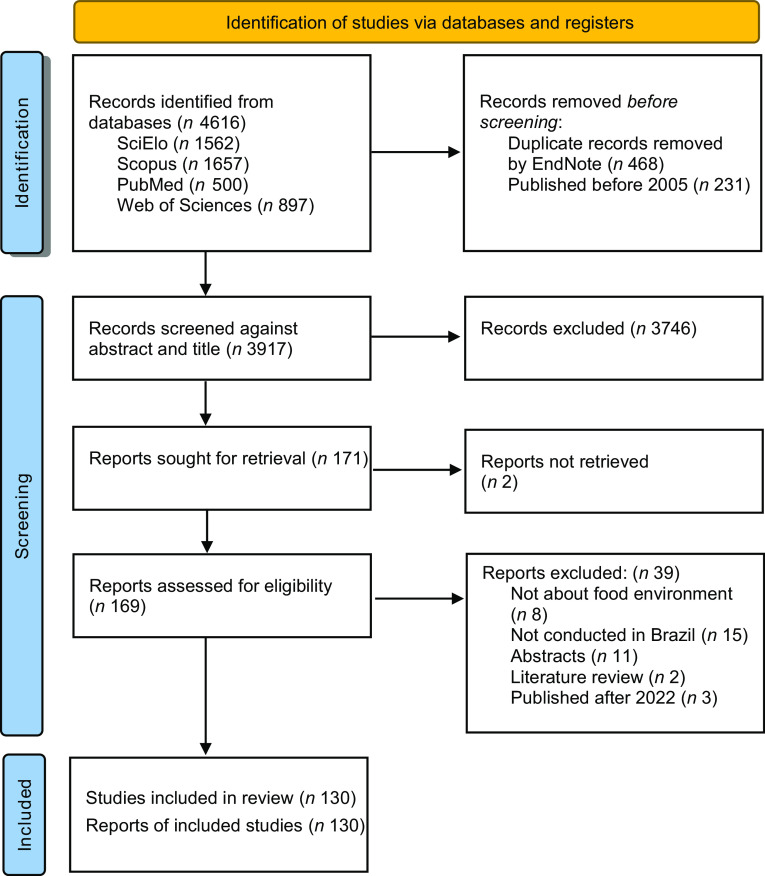
PRISMA-ScR flow diagram of the selection process

The search strategy resulted in 4616 articles. After removing duplicates (*n* 468) and publications before 2005 (*n* 231), 3917 unique records were identified, and 171 of them were considered for full-text screening against eligibility criteria. The agreement rate between the two reviewers was 99·1 %. In the end, 130 articles were included (Fig. 1). The complete list of articles with a description of the main characteristics of the articles is provided in Supplementary Material S2.

**Fig. 2:**
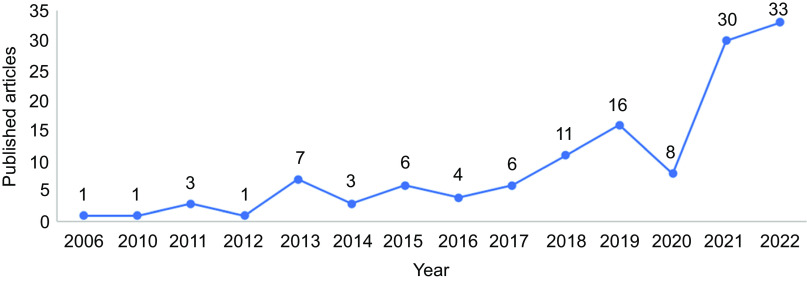
Number of studies on Brazilian food environments published per year (*n* 130)

There was a significant increase in the number of articles published per year as of 2018, especially from 2020 to 2022. The peak in the number of publications occurred in 2022 (25·4 %) (Fig. 2). Among the 112 studies included, the first author of 128 articles had an institutional affiliation with Brazilian universities or research institutions. The institutions that appeared most frequently in the affiliation of the first author are the Federal University of Minas Gerais (UFMG) (26·9 %) and the University of São Paulo (USP) (23·1 %), both from the Southeast region of Brazil (data not shown).

Geographically, research on the food environment in Brazil is mostly concentrated in the Southeast region (53·7 %), in state capitals (56·8 %) and in metropolitan regions (22·9 %). The number of studies carried out in the North, Northeast and Central-West regions (24·5 %), together, was similar to that of works referring to the South region individually (17·6 %). In addition, only twenty-four articles (20·3 %) analysed food environment scenarios in small- or medium-sized cities. Regarding the language of publication, ninety-eight articles are available in English (75·4 %), twenty both in English and Portuguese (15·4 %), and twelve in Portuguese (9·2 %) (Table 1). No full text was available in Spanish.

**Table 1:** General characteristics of studies on food environments in Brazil included in the present review

Characteristics of the studies	Frequency	
	*n*	%
Studies included (total)	130	100·0
Macroregion*		
Southeast	101	53·7
South	33	17·6
Northeast	19	10·1
North	15	8·0
Central-West	12	6·4
Uninformed	1	0·5
Not applicable	7	3·7
Geographic coverage*		
State capitals	84	56·8
Metropolitan regions	34	22·9
Countryside	30	20·3
Language of publication		
English	98	75·4
English and Portuguese	20	15·4
Portuguese	12	9·2
Year of publication		
2005–2010	2	1·5
2011–2016	24	18·5
2017–2019	33	25·4
2020–2022	71	54·6
Focus on food environments		
Primary	105	80·8
Secondary	25	19·2
Study design		
Cross-sectional	82	63·1
Ecological	27	20·8
Methodological (validity or reliability of instruments and measures)	11	8·4
Commentary or opinion	5	3·8
Community trial	3	2·3
Cohort	1	0·8
Development of conceptual model	1	0·8
Methodological approach		
Quantitative analytical	68	52·3
Quantitative descriptive	53	40·8
Qualitative	3	2·3
Not applicable	6	4·6

* The same article can be included in more than one category.

It is noteworthy that for 80·8 % of the studies, the analysis of food environments was their main focus, while the others secondarily investigated some food environment-related variables. A quantitative analytical approach was used in sixty-eight studies (52·3 %), while only three (2·3 %) had a qualitative approach. The predominant epidemiological study design was cross-sectional (63·1 %), both among descriptive studies (*n* 26) and among analytical studies (*n* 55), followed by the ecological design (20·8 %, twenty-one were descriptive and six analytical), and studies of validity or reliability of instruments and measures (8·4 %) (Table 1).

The community food environment was the main type evaluated in Brazil (52·7 %), followed by the organisational environment (26·7 %), with emphasis on the investigation of the school environment (*n* 26), and the consumer food environment (13·0 %). It is noteworthy that the articles that addressed the digital food environment (3·5 %) were published as of 2020. Almost all of the studies (66·7 %) addressed the physical food environment, and 22·9 % covered the sociocultural dimension (Table 2).

**Table 2:** Methodological aspects of studies on the food environment in Brazil included in the present review (*n* 130)

Characteristics of the studies	Frequency	
	*n*	%
Type of food environment*		
Community	77	52·7
Organisational	39	26·7
Consumer	19	13·0
Information	6	4·1
Digital	5	3·5
Dimension of food environment*		
Physical	128	66·7
Sociocultural	44	22·9
Economic	13	6·8
Policy	7	3·6
Population group*		
Adults	37	26·5
Adolescents	20	14·3
Schoolchildren	14	10·0
Elderly	7	5·0
Pregnant and/or lactating women	3	2·1
Preschool children	1	0·7
Not applicable	58	41·4
Sampling plan (individuals) (*n* 71)		
Representative sample	54	76·1
Convenience sample	16	22·5
Census	1	1·4
Sampling plan (other unit of analyses) (*n* 52)		
Representative sample	15	28·8
Convenience sample	15	28·8
Census	22	42·4
Data source		
Primary	70	53·8
Secondary	43	33·1
Primary and secondary	10	7·7
Not applicable	7	5·4
Conceptual models adopted*		
Not clearly mentioned	101	75·9
Glanz et al. (2005)†	18	13·4
Swinburn et al. (1999)‡	4	3·0
FAO (2019)§	2	1·5
Granheim et al. (2020)||	2	1·5
Caspi et al. (2012)**	2	1·5
Gálvez-Espinoza et al. (2017)¶	1	0·8
Granheim (2019)††	1	0·8
Story et al. (2008)‡‡	1	0·8
Swinburn et al. (2013)§§	1	0·8
Outcomes or aspects assessed*		
Food consumption	29	27·1
Description of the environment	29	27·1
Parameters for adiposity measurement	20	18·7
Validation and/or assessment of the reliability of instruments	10	9·3
Non-communicable diseases	5	4·7
Availability and affordability	4	3·8
School feeding	3	2·8
Physical environment	3	2·8
Physical activity	2	1·9
Food acquisition	1	0·9
Gestational weight gain	1	0·9

* The same article can be included in more than one category.

† Glanz K, Sallis JF, Saelens BE et al. (2005) Healthy nutrition environments: concepts and measures. Am J Health Promot 19, 5, 330–3. doi: 10·4278/0890–1171–19·5·330.

‡ Swinburn B, Egger G, Raza F (1999) Dissecting obesogenic environments: the development and application of a framework for identifying and prioritising environmental interventions for obesity. Prev Med 29, 6, 563–570.

§ Food and Agriculture Organization of the United Nations (FAO) (2019) School food and nutrition framework. Rome: FAO.

|| Granheim SI, Opheim E, Terragni L et al. (2020) Mapping the digital food environment: a scoping review protocol. BMJ Open 10, 4, e036241. doi: 10·1136/bmjopen-2019–036 241.

¶ Espinoza PG, Egaña D, Masferrer D et al. (2018) Propuesta de un modelo conceptual para el estudio de los ambientes alimentarios en Chile. Rev Panam Salud Publica 41, e169. doi: 10·26 633/RPSP.2017·169.

** Caspi CE, Sorensen G, Subramanian SV et al. (2012) The local food environment and diet: a systematic review. Health Place 18, 5, 1172–87. doi: 10·1016/j.healthplace.2012·05·006.

†† Granheim SI (2019) The digital food environment. UNSCN Nutrition 44, 116–121.

‡‡ Story M, Kaphingst KM, Robinson-O’Brien R et al. (2008) Creating healthy food and eating environments: Policy and environmental approaches. Annu Rev Public Health 29, 1, 253–272. doi: 10·1146/annurev.publhealth.29·020 907·090 926.

§§ Swinburn B, Sacks G, Vandevijvere S et al. (2013) INFORMAS (International Network for Food and Obesity/non-communicable diseases Research, Monitoring and Action Support): overview and key principles. Obes Rev 14, 1, 1–12.

Seventy-one studies analysed the food environment related to some population groups. Most of them covered the adult population (26·5 %), adolescents (14·3 %), or schoolchildren (10·0 %), and 76·1 % of those evaluated a representative sample of the population, and 22·5 % used a convenience sample. Among fifty-two studies that investigated other units of analysis (e.g. retail and markets), 42·4 % carried out a census, 28·8 % analysed a representative sample and 28·8 % analysed a convenience sample. Regarding the outcome of the studies, the most recurrent were food consumption (27·1 %), description of the environment (27·1 %), parameters for adiposity measurement (18·7 %), and validation and/or assessment of the reliability of instruments for measuring the environment (9·3 %) (Table 2).

In 101 articles, the adoption of a conceptual model on the food environment was not clearly mentioned. Among the studies that explained the adoption of a model, the most cited was that of Glanz et al. (2005) (13·4 %). Only four were based on the model proposed by Swinburn et al. (1999), and two or fewer studies were based on other models (Table 2).

There was a wide range of instruments for the measurement of food environments in Brazil. Most studies used instruments designed particularly for the collection of food environment data (*n* 24) or analysed variables collected in questionnaires applied in national surveys (*n* 16), for example, the National Survey of School Health (PeNSE)^(17–19)^ and the Study of Cardiovascular Risks in Adolescents (ERICA)^(20,21)^. The instruments of the Obesogenic Environment Study in São Paulo (ESAO)^(22)^ (*n* 9), adapted versions of the Nutrition Environment Measurement Tool (NEMS)^(23)^ (*n* 8) and the Audit-NOVA^(24)^ (*n* 7) (data not shown) were also relevant.

The main limitation pointed out by the authors of food environment studies was the possibility of bias in data collection (19·4 %), the use of secondary data (18·5 %) and the lack of a representative sample (17·1 %). Other limitations reported included old data (11·1 %) and do not evaluate informal markets (8·5 %) (Table 3).

**Table 3:** Limitations pointed out by the authors concerning data on food environments from the Brazilian scientific articles included in the present review (*n* 130)

Limitations*	Frequency	
	*n*	%
Possible bias in data collection	25	19·4
Secondary data	24	18·5
Non-representativeness of the population	22	17·1
Old data	15	11·6
Do not evaluate informal markets	11	8·5
Euclidean buffer	9	7·0
Do not evaluate all establishments	9	7·0
Classification of establishments	7	5·4
Possibility of errors in georeferencing	4	3·1
Instrument not validated	2	1·6
Euclidean distance	1	0·8

* The same article can be included in more than one category.

## Discussion

The present scoping review evaluated the scientific research on the food environment in Brazil and identified 130 studies carried out between January 2005 and December 2022. Most of them were conducted in some capital cities of the Southeast region. The analytical quantitative approach and the cross-sectional design were the most frequently used. Most articles were published in English. Most studies assessed the community food environment and focused on aspects of the physical dimension; most often, they addressed the adult population and used primary data. In addition, most of the articles did not use a formal definition of the food environment according to a given conceptual model. They described the food environment and adopted food consumption as the main outcome.

The growing number of publications on food environments in Brazil in recent years and the predominance of English as the language of publication shows that Brazilian scientific research on this topic has been intense and is accessible to the global scientific community. These results contrast with the report by Turner and colleagues in 2018 and 2020^(3,7)^, which highlighted the immaturity and low quality of research on food environments in low- and middle-income countries, such as Brazil. Such claims may have been made as a result of a restricted search strategy, as pointed out by Mendes et al.^(25)^.

Articles differ widely in terms of region and geographic location. This scenario is, to some extent, convergent with regional inequalities in science, technology and innovation in Brazil. The two universities with the highest production of research on food environments (UFMG and USP) are among the top universities in the country in terms of scientific research^(26)^. They are located in states of the Southeast region, which receives most funds for research^(27,28)^, and their major *campuses* were built in capital cities (Belo Horizonte and São Paulo). On the other hand, of the total number of Brazilian municipalities (*n* 5570), the vast majority are located in the inland towns of the states and there is a great economic, sociocultural and urban development diversity among them^(29)^. This points to the need to conduct food environment research in Brazil’s countryside in order to encourage the formulation of public policies that take into account different realities and scenarios.

Despite the community food environment being assessed in half of the studies evaluated (*n* 52·7 %), the discussion about food deserts and swamps is relatively new in Brazil, with only six articles measuring it and the first one published in 2017^(30)^. At least in part, the incorporation of these analyses in the scientific literature could be induced by the Technical study – mapping food deserts in Brazil^(31)^, a publication made by the Ministry of Social Development in 2018. It can be considered a good example of the mutual influence of government and the academy, with scientific evidence orienting the public policies but also decision-makers influencing the research agenda.

Most of the articles used primary data. However, in one-third of them, the instrument used to measure the environment was particularly designed for the study itself, often without validity and reliability assessment. This casts doubt on the quality of the data and the interpretation of the findings; also, it hinders reproducibility and comparability with other studies. However, it is worth mentioning that this weakness is not found only in Brazilian studies. A systematic review of food environment measurement found that less than 30 % of studies published up to 2015 reported the validity and reliability of the measures^(6)^. Therefore, it is worth highlighting the relevance of the development of the Audit-NOVA^(24)^, a reliable instrument to assess the food environment of consumers in Brazil based on the NOVA classification system^(32)^ and the recommendations of the Brazilian Dietary Guidelines^(33)^.

More than half of the studies analysed in the present review did not present a formal definition of the food environment that had been underpinned by any existing conceptual models, nor was their design based on any of them. To some extent, this may imply that study design in this field is still incipient and may have repercussions on the conception of procedures for measuring food environments. Among those which relied on a model, most were based on that of Glanz et al.^(14)^. This is due to the fact that it is one of the first conceptual models published, and its approach is researcher-friendly. Also, it is compatible with the NEMS as a measurement instrument and with instruments developed on the basis of NEMS, for example, the ESAO^(22)^. It is certainly important to adopt conceptual models in future studies, especially considering the important advances found in models proposed more recently, for example, the inclusion of sustainability and the clarification of the informal market^(4)^, the recognition of the interconnection between different food environments^(34)^, the in-depth description of the constituent elements of organisational environments^(35)^, and the recognition of the material impact of digital environments on physical environments^(15)^.

Despite the evaluation of the quality of the studies is not expected in scoping reviews, the limitations pointed out by authors and recognised in the peer review process were mapped in other to dialogue with the quality assessment but mainly to contribute with elements that can influence the research agenda and the planning of new studies in the field. Limitations of food environment data, as pointed out by authors, include the possibility of bias in data collection (e.g. owing to the use of non-validated questionnaires) and the use of non-representative data (e.g. resulting from the use of convenience samples for the population), as well as the use of secondary data as a source of food environment data.

If, on the one hand, data not particularly collected for use in scientific research may not have important information, on the other hand, such data often have a greater scope, even different or additional information, and, in some cases, are representative of the context of analysis. This is less common in studies with primary data^(36,37)^. Furthermore, much of the Brazilian public administrative data, at national and local levels, is open; therefore, it can be more readily used for the conduction of different types of studies. In this sense, this type of information source, which is available in Brazil, a low- and middle-income country, is particularly relevant. It is crucial to expand and improve the content and quality of these databases, not only for administrative purposes but also to promote the advancement of research on the food environment in Brazil. There should be further analyses of the validity of secondary databases. For places that have small territories, for example, urban areas of inland towns, direct observation can be an alternative for the validation of secondary databases, and they could also be the gold standard for the collection of food environment data^(38)^. In addition, data can be validated using two secondary databases, and the discrepancy between them could be evaluated through direct observation in places with small territories, or through virtual conferencing, as is the case of the Brazilian study by Rocha et al.^(37)^, or using virtual tools that allow the visualisation of streets, for example, Google Street View^(38–41)^.

A strong point of this review is that the search strategy did not use terms restricted to the jargon of a specific conceptual model; therefore, the researchers could identify studies based on different concepts and terminologies of the food environment. As for limitations, some authors suggest that the search strategy of a scoping review should be comprehensive in order to identify both published evidence and grey literature because unpublished studies are potentially relevant^(42)^. However, this is not a requirement of the PRISMA-ScR guidelines^(13)^, and a decision was made for the present review to focus on published and peer-reviewed articles. In the absence of a basis for registering scoping review protocols, the existing alternative is to publish the protocol as a scientific article. However, only a limited number of journals accept this publication format. The present researchers chose not to do so, but it should be noted that this is also not a PRISMA-ScR prerequisite^(13)^.

We expect this scoping review contributes to the discussion about the need for diversity in the research. Different from reviews conducted by North Global researchers, it has been observed a significant potential for research from Latin American countries^(12)^ and the production of knowledge with varied perspectives, not only replications of studies from high-income countries, can be strategic to change and tailored the research agenda, but also public policies.

Conceptual models developed by researchers from countries like Chile^(5)^ and Brazil^(35)^ can inspire news studies on food environments through their application and the development of others, specific for some realities or covering some lacks. An interesting example of a Global South initiative was the establishment of The Africa Food Environment Research Network (FERN), with the following objectives: ‘(1) building research capacity for innovative food environment research in Africa; (2) improving South–South and South–North partnerships to stimulate robust food environment research and monitoring in Africa; and (3) sustaining dialogue and focusing priorities around current and future needs for enhanced food environment research and monitoring in Africa’^(43)^. Despite all of this, the research conducted in and the researchers based on low- and middle-income countries and the Global South should be valued and received adequate funding to minimise global inequalities.

## Conclusion

Literature on the food environment in Brazil has expanded in recent years, as a sign of growing recognition of the potential impact of publishing research information on food practices and human health. The present scoping review mapped this emerging scientific research in the country, mainly available in English, identifying the concentration of the cross-sectional studies in the richest region, conducted by researchers from a few universities and focusing on the community food environment.

The review has the potential to contribute to the work of researchers by pointing out particular gaps, for example, to conduct studies on the food environment in the Brazilian countryside, to support the formulation of research questions in conceptual models, to use valid and reliable instruments to collect primary data, and to recognise the importance of conducting more longitudinal, interventional and qualitative studies. The consolidation and advancement of this research agenda can potentially provide further insights into food environments, their configuration in low- and middle-income countries, and their influence on food practices and health outcomes, as well as into the formulation of interventions and better management of public policies.

## Supplementary material

For supplementary material accompanying this paper visit https://doi.org/10.1017/S1368980023000836. click here to view supplementary material

## References

[1] Swinburn BA , Kraak VI , Allender S et al. (2019) The global syndemic of obesity, undernutrition, and climate change: the Lancet Commission report. Lancet 393, 791–846. doi: 10.1016/S0140-6736(18)32822-8. 30700377

[2] HLPE (2017) *A Report by the High Level of Experts on Food Security and Nutrition of the Committee on World Food Security*. Rome: FAO.

[3] Turner C , Aggarwal A , Walls H et al. (2018) Concepts and critical perspectives for food environment research: a global framework with implications for action in low- and middle-income countries. Glob Food Sec 18, 93–101. doi: 10.1016/j.gfs.2018.08.003.

[4] Downs SM , Ahmed S , Fanzo J et al. (2020) Food environment typology: advancing an expanded definition, framework, and methodological approach for improved characterization of wild, cultivated, and built food environments toward sustainable diets. Foods 9, 532. doi: 10.3390/foods9040532. 32331424 PMC7230632

[5] Caspi CE , Sorensen G , Subramanian SV et al. (2012) The local food environment and diet: a systematic review. Health Place 18, 1172–1187. doi: 10.1016/j.healthplace.2012.05.006. 22717379 PMC3684395

[6] Lytle LA & Sokol RL (2017) Measures of the food environment: a systematic review of the field, 2007–2015. Health Place 44, 18–34. 28135633 10.1016/j.healthplace.2016.12.007

[7] Turner C , Kalamatianou S , Drewnowski A et al. (2020) Food environment research in low- and middle-income countries: a systematic scoping review. Adv Nutr 11, 387–397. doi: 10.1093/advances/nmz031. 31079142 PMC7442349

[8] Muzenda T , Dambisya PM , Kamkuemah M et al. (2022) Mapping food and physical activity environments in low- and middle-income countries: a systematised review. Health Place 75, 102809. doi: 10.1016/j.healthplace.2022.102809. 35508088

[9] Toure D , Herforth A , Pelto GH et al. (2021) An emergent framework of the market food environment in low- and middle-income countries. Curr Dev Nutr 5, 4. doi: 10.1093/cdn/nzab023. PMC8075774 33948531

[10] Black C , Ntani G , Inskip H et al. (2014) Measuring the healthfulness of food retail stores: variations by store type and neighbourhood deprivation. Int J Behav Nutr Phys Act 11, 69. 24884529 10.1186/1479-5868-11-69 PMC4132210

[11] Mackenbach JD , Rutter H , Compernolle S et al. (2014) Obesogenic environments: a systematic review of the association between the physical environment and adult weight status, the SPOTLIGHT project. BMC Public Health 14, 233. 24602291 10.1186/1471-2458-14-233 PMC4015813

[12] Pérez-Ferrer C , Auchincloss AH , de Menezes MC et al. (2019) The food environment in Latin America: a systematic review with a focus on environments relevant to obesity and related chronic diseases. Public Health Nutr 22, 3447–3464. doi: 10.1017/S1368980019002891. 31666140 PMC10260576

[13] Tricco AC , Lillie E , Zarin W et al. (2018) PRISMA extension for scoping reviews (PRISMA-ScR): checklist and explanation. Ann Intern Med 169, 467–473. doi: 10.7326/M18-0850. 30178033

[14] Glanz K , Sallis JF , Saelens BE et al. (2005) Healthy nutrition environments: concepts and measures. Am J Health Promot 19, 330–333. doi: 10.4278/0890-1171-19.5.330. 15895534

[15] Granheim SI (2019) The digital food environment. UNSCN Nutr 44, 116–121.

[16] Swinburn B , Sacks G , Vandevijvere S et al. (2013) INFORMAS (International Network for Food and Obesity/non-communicable diseases Research, Monitoring and Action Support): overview and key principles. Obes Rev 14, 1–12. 10.1111/obr.12087 24074206

[17] IBGE (2013) National School Health Survey: 2012. Rio de Janeiro: IBGE.

[18] IBGE (2016) National School Health Survey: 2015. Rio de Janeiro: IBGE.

[19] IBGE (2021) National School Health Survey: 2019. Rio de Janeiro: IBGE.

[20] Bloch KV , Szklo M , Kuschnir MCC et al. (2015) The study of cardiovascular risk in adolescents–ERICA: rationale, design and sample characteristics of a national survey examining cardiovascular risk factor profile in Brazilian adolescents. BMC Public Health 15, 1–10. doi: 10.1186/1471-2458-15-1. 25880653 PMC4334602

[21] Silva TLN , Klein CH , Souza AM et al. (2016) Response rate in the study of cardiovascular risks in adolescents–ERICA. Rev Saude Publica 50, 1s–13s. doi: 10.1590/s01518-8787.2016050006730. PMC4767034 26910552

[22] Duran AC , Lock K , Latorre MRDO et al. (2015) Content validity and reliability of a university food environment assessment instrument. Rev Saúde Pública 49, 80.26538101

[23] Martins PA , Cremm EC , Leite FH et al. (2013) Validation of an adapted version of the nutrition environment measurement tool for stores (NEMS-S) in an urban area of Brazil. J Nutr Educ Behav 45, 785–792. doi: 10.1016/j.jneb.2013.02.010. 23747064

[24] Borges CA & Jaime PC (2019) Development and evaluation of food environment audit instrument: AUDITNOVA. Rev Saúde Pública 53, 91. doi: 10.11606/s1518-8787.2019053001316. 31644722 PMC9586434

[25] Mendes LL , Pessoa MC & Duarte CK (2020) Comments on the article: “Food Environment Research in Low- and Middle-Income Countries: A Systematic Scoping Review”. Adv Nutr 11, 1044–1045. doi: 10.1093/advances/nmaa018. 32666112 PMC7360444

[26] CWTS (2022) CWTS Leiden Ranking 2021. https://www.leidenranking.com/ranking/2021/list (accessed June 2022).

[27] Cavalcante LR (2022) Regional Inequalities In Science, Technology and Innovation (ST&I) in Brazil: An Analysis of Its Recent Evolution. Rio de Janeiro: IPEA.

[28] Silva KKRB & Soares SV (2021) The characterization of State Research Support Foundations. XX Colóquio Internacional de Gestão Universitária. https://repositorio.ufsc.br/handle/123456789/230275 (accessed June 2022).

[29] Santos JAF (2018) Social class, territory and health inequality in Brazil. Saude Soc 27, 556–572. doi: 10.1590/S0104-12902018170889.

[30] Davies G , Frausin G & Parry L (2017) Are there food deserts in rainforest cities? Ann Assoc Am Geogr 107, 794–811. doi: 10.1080/24694452.2016.1271307.

[31] Interministerial Chamber of Food Security and Nutrition (2018) Mapping food deserts in Brazil. https://aplicacoes.mds.gov.br (accessed March 2023).

[32] Monteiro CA , Cannon G , Levy R et al. (2016) NOVA. The star shines bright. World Nutr 7, 1–3; 28–38.

[33] Brazil, Ministry of Health of Brazil & Department of Health Care & Primary Health Care Department (2014) Dietary Guidelines for the Brazilian Population. Brasilia: Ministry of Health of Brazil.

[34] Espinoza PG , Egaña D , Masferrer D et al. (2018) Proposal for a conceptual model for the study of food environments in Chile. Rev Panam Salud Publica 41, e169. doi: 10.26633/RPSP.2017.169. PMC6650624 31384280

[35] Castro IRR & Canella DS (2022) Organizational food environments: advancing their conceptual model. Foods 11, 993. doi: 10.3390/foods11070993. 35407080 PMC8998120

[36] Menezes MC , de Matos VP , de Pina MF et al. (2021) Web data mining: validity of data from Google earth for food retail evaluation. J Urban Health 98, 285–295. 33230671 10.1007/s11524-020-00495-x PMC8079479

[37] Rocha LL , Carmo AS , Jardim MZ et al. (2023) The community food environment of a Brazilian metropolis. Food Cult Soc 26, 182–192. doi: 10.1080/15528014.2021.1987027.

[38] Lucan SC (2015) Concerning limitations of food-environment research: a narrative review and commentary framed around obesity and diet-related diseases in youth. J Acad Nutr Diet 115, 205–212. doi: 10.1016/j.jand.2014.08.019. 25443565

[39] Pliakas T , Hawkesworth S , Silverwood RJ et al. (2017) Optimising measurement of health-related characteristics of the built environment: comparing data collected by foot-based street audits, virtual street audits and routine secondary data sources. Health Place 43, 75–84. doi: 10.1016/j.healthplace.2016.10.001. 27902960 PMC5292100

[40] Liese ADN , Colabianchi AP , Lamichhane TL et al. (2010) Validation of 3 food outlet databases: completeness and geospatial accuracy in rural and urban food environments. Am J Epidemiol 172, 1324–1333. doi: 10.1093/aje/kwq292. 20961970 PMC2991633

[41] Rundle AG , Bader MD , Richards CA et al. (2011) Using Google Street View to audit neighborhood environments. Am J Prev Med 40, 94–100. doi: 10.1016/j.amepre.2010.09.034. 21146773 PMC3031144

[42] Peters MD , Godfrey CM , Khalil H et al. (2015) Guidance for conducting systematic scoping reviews. Int J Evid Based Healthc 13, 141–146. 26134548 10.1097/XEB.0000000000000050

[43] Tandoh A , Aryeetey R , Agyemang C et al. (2022) The Africa Food Environment Research Network (FERN): from concept to practice. Glob Health Promot 0, 0. doi: 10.1177/17579759221126155. 36321592

