# Supplementary material for: Scientific research on food environments in Brazil: a scoping review
Source: Public Health Nutr. 2023 May 26;26(10):2056–65. doi: 10.1017/S1368980023000836 (PMC10564610; doi:10.1017/S1368980023000836)
Supplement: Supplementary file 1 [file S1368980023000836sup.zip › S1368980023000836sup001.docx]

**Supplementary Material S1.** Search strategy for the study Scientific research on food environments in Brazil: a scoping review

Search strategy: PubMed and Web of Science

((“Brasil” OR “Brazil”) AND ((“food environment” OR “food environments” OR “nutrition environment” OR “nutrition environments” OR “food environment”) AND (“community” OR “organizational” OR “consumer” OR “information” OR “home” OR “school” OR “digital” OR “virtual” OR “perceived” OR “observed” OR “neighborhood” OR “retail” OR “local” OR “urban” OR “natural” OR “built” OR “formal” OR “informal” OR “university” OR “hospital” OR “workplace”)) OR (“food swamp” OR “food swamps” OR “food desert” OR "Food Deserts"[Mesh] OR “food deserts”))

Search strategy: Scopus

((‘Brasil’ OR ‘Brazil’) AND ((‘food environment’ OR ‘food environments’ OR ‘nutrition environment’ OR ‘nutrition environments’ OR ‘food environment’) AND (‘community’ OR ‘organizational’ OR ‘consumer’ OR ‘information’ OR ‘home’ OR ‘school’ OR ‘digital’ OR ‘virtual’ OR ‘perceived’ OR ‘observed’ OR ‘neighborhood’ OR ‘retail’ OR ‘local’ OR ‘urban’ OR ‘natural’ OR ‘built’ OR ‘formal’ OR ‘informal’ OR ‘university’ OR ‘hospital’ OR ‘workplace’)) OR (‘food swamp’ OR ‘food swamps’ OR ‘food desert’ OR ‘food deserts’))

Search strategy: Scielo

((((food environment) OR (food environments) OR (nutrition environment) OR (nutrition environments) OR (food environment)) AND ((community) OR (organizational) OR (consumer) OR (information) OR (home) OR (school) OR (digital) OR (virtual) OR (perceived) OR (observed) OR (neighborhood) OR (retail) OR (local) OR (urban) OR (natural) OR (built) OR (formal) OR (informal) OR (university) OR (hospital) OR (workplace))) OR ((food swamp) OR (food swamps) OR (food desert) OR (Food Deserts)))
