# Supplementary material for: Scientific research on food environments in Brazil: a scoping review
Source: Public Health Nutr. 2023 May 26;26(10):2056–65. doi: 10.1017/S1368980023000836 (PMC10564610; doi:10.1017/S1368980023000836)
Supplement: Supplementary file 1 [file S1368980023000836sup.zip › S1368980023000836sup002.docx]

**Supplementary Material S2.** Description of the articles included in the scoping review on scientific research on food environments in Brazil (n=130).

| **First author**  **(year of publication)** | **Institution affiliation of the first author** | **Journal name** | **Language** | **Study design** | **Methodological approach** | **Macroregion where the study was conducted** | **Geographic coverage** | **Data source** | **Study population** | **Type of food environment** | **Dimension of the food environment** | **Adoption of conceptual food environment models** |
| --- | --- | --- | --- | --- | --- | --- | --- | --- | --- | --- | --- | --- |
| Albuquerque (2022)^128^ | Universidade Federal de Viçosa | American Journal of Human Biology | English | Cross-sectional | Quantitative analytical | Southeast | Countryside | Primary | School children | Community | Physical | No |
| Albuquerque (2022)^129^ | Universidade Federal de Viçosa | American Journal of Human Biology | English | Cross-sectional | Quantitative analytical | Southeast | Countryside | Primary | School children | Organizational | Physical | No |
| Almeida (2021a)¹ | Universidade Federal de Minas Gerais | Public Health Nutrition | English | Cross-sectional | Quantitative analytical | Southeast | Countryside | Primary, Secondary | Elderly | Community | Physical | No |
| Almeida (2021b)² | Universidade Federal de Minas Gerais | Journal of the American College of Nutrition | English | Ecological | Quantitative analytical | Southeast | Countryside | Primary | - | Community | Sociocultural, Physical | No |
| Almeida (2018)³ | Universidade de São Paulo | Public Health Nutrition | English | Cross-sectional | Qualitative | Southeast | State capitals | Primary | Adults | Community | Economic, Sociocultural, Physical | No |
| Almeida (2022)^123^ | Universidade Federal de Ciências da Saúde de Porto Alegre | British Journal of Nutrition | English | Cross-sectional | Quantitative analytical | South | State capitals | Primary | Adults, Elderly | Community | Physical | No |
| Alves (2019)^4^ | Universidade Federal de Santa Catarina | International Journal of Environmental Research and Public Health | English | Cross-sectional | Quantitative analytical | South | State capitals | Primary | School children, Adolescents | Community | Physical | No |
| Andretti (2021)^5^ | Fundação Getúlio Vargas | Preventive Medicine | English | Cohort | Quantitative analytical | - | - | Secondary | School children, Adolescents | Organizational | Economic | No |
| Araújo (2022)^6^ | Universidade Federal de Ouro Preto | Ciência & Saúde Coletiva | Portuguese | Ecological | Quantitative descriptive | Southeast | Metropolitan regions | Primary | - | Community | Sociocultural, Physical | No |
| Assis (2018)^7^ | Universidade Federal de Juiz de Fora | Revista Paulista de Pediatria | English, Portuguese | Cross-sectional | Quantitative analytical | Southeast | Countryside | Primary | School children, Adolescents | Community | Physical , Sociocultural | No |
| Assis (2019)^8^ | Universidade Federal de Juiz de Fora | Public Health Nutrition | English | Cross-sectional | Quantitative analytical | Southeast | Countryside | Primary, Secondary | School children, Adolescents | Community | Sociocultural, Physical | No |
| Assis (2022)^130^ | Universidade Federal de Minas Gerais | BMC Public Health | English | Cross-sectional | Quantitative analytical | Southeast | State capitals | Secondary | Adolescents | Organizational | Physical | No |
| Azeredo (2016)^9^ | Universidade Federal de Uberlândia | Preventive Medicine | English | Cross-sectional | Quantitative analytical | North, South, Northeast, Southeast, Central-West | State capitals, Metropolitan regions, Countryside | Secondary | Adolescents | Organizational, Community | Physical | No |
| Azeredo (2020)^10^ | Universidade Federal de Uberlândia | Revista de Saúde Pública | English | Cross-sectional | Quantitative analytical | North, South, Northeast, Southeast, Central-West | State capitals | Secondary | Adolescents | Organizational | Physical , Policy, Sociocultural | No |
| Backes (2019)^11^ | Universidade do Vale do Rio dos Sino | Cadernos de Saúde Pública | English | Cross-sectional | Quantitative analytical | South | Metropolitan regions | Primary | Adults | Community | Physical , Sociocultural | No |
| Backes (2021)^12^ | Prefeitura de São Leopoldo | Ciência & Saúde Coletiva | Portuguese | Ecological | Quantitative descriptive | South | Metropolitan regions | Primary, Secondary | - | Community, Consumer | Economic, Sociocultural, Physical | No |
| Bandoni (2021)^13^ | Universidade de São Paulo | Health Behavior and Policy Review | English | Cross-sectional | Quantitative analytical | North, South, Northeast, Southeast, Central-West | State capitals , Metropolitan regions, Countryside | Secondary | School children | Organizational | Physical | No |
| Bandoni (2011)^14^ | Universidade de São Paulo | Public Health Nutrition | English | Community trial | Quantitative analytical | Southeast | State capitals | Primary | Adults | Organizational | Physical | No |
| Bandoni (2010)^15^ | Universidade de São Paulo | Nutrition & Food Science | English | Cross-sectional | Quantitative analytical | Southeast | State capitals | Primary | Adults | Organizational | Physical , Sociocultural | No |
| Barbosa (2020)^16^ | Universidade Federal Fluminense | Revista Chilena de Nutrición | English | Cross-sectional | Quantitative descriptive | Southeast | Metropolitan regions | Primary | - | Organizational | Physical | Glanz et al (2005)^113^ |
| Barbosa (2022)^124^ | Universidade Estadual do Ceará | Revista de Nutrição | English | Ecological | Quantitative descriptive | South | State capitals | Secondary | - | Community | Physical | No |
| Batista (2022)^125^ | Universidade de São Paulo | Ciência & Saúde Coletiva | English, Portuguese | Cross-sectional | Quantitative analytical | Southeast | State capitals, Metropolitan regions | Primary | - | Information | Physical | No |
| Borges (2019)^17^ | Universidade de São Paulo | Revista de Saúde Pública | English, Portuguese | Studies of validity or reliability of instruments and measures | Quantitative descriptive | Southeast | Metropolitan regions | Primary | - | Consumer | Physical | Glanz et al (2005) ^113^ |
| Borges (2022)^18^ | Universidade de São Paulo | Cadernos de Saúde Pública | English, Portuguese | Ecological | Quantitative analytical | Southeast | Metropolitan regions | Primary | - | Community | Physical | No |
| Borges (2018)^19^ | Universidade de São Paulo | Sustainability | English | Ecological | Quantitative descriptive | Southeast | Metropolitan regions | Primary | - | Consumer, Community | Sociocultural, Physical | Glanz et al (2005) ^113^ |
| Borges (2021)^20^ | Universidade de São Paulo | International Journal of Environmental Research and Public Health | English | Studies of validity or reliability of instruments and measures | Quantitative analytical | Southeast | Metropolitan regions | Primary | - | Consumer | Economic, Physical | Glanz et al (2005) ^113^ |
| Botelho (2020)^21^ | Escola Nacional de Saúde Pública Sérgio Arouca | Cadernos de Saúde Pública | English, Portuguese | Commentary or Opinion | - | - | - | - | - | Digital | Physical | Granheim (2019)114 |
| Camargo (2019)^22^ | Universidade Estadual de Campinas | Cadernos de Saúde Pública | English | Cross-sectional | Quantitative descriptive | Southeast | Metropolitan regions | Primary, Secondary | Adults | Community | Sociocultural, Physical | No |
| Carmo (2018)^23^ | Universidade Federal de Minas Gerais | Cadernos de Saúde Pública | English | Ecological | Quantitative descriptive | North, South, Northeast, Southeast, Central-West | State capitals , Metropolitan regions, Countryside | Secondary | - | Organizational, Community | Physical | No |
| Castro (2022)^24^ | Universidade do Estado do Rio de Janeiro | Foods | English | Development of conceptual model | - | - | - | - | - | Organizational | Physical , Policy | Gálvez-Espinoza et al (2017)^115^ |
| Chor (2016)^25^ | Escola Nacional de Saúde Pública Sérgio Arouca | BMC Public Health | English | Cross-sectional | Quantitative analytical | South, Northeast, Southeast | State capitals | Secondary | Adults, Elderly | Community | Physical | No |
| Cordeiro (2022)^126^ | Universidade Federal de Minas Gerais | Journal of Hunger & Environmental Nutrition | English | Cross-sectional | Quantitative analytical | Southeast | State capitals | Primary | - | Community | Physical | No |
| Corrêa (2018a)^26^ | Universidade Federal de Santa Catarina | Journal of Public Health | English | Cross-sectional | Quantitative analytical | South | State capitals | Secondary | School children | Organizational | Physical | No |
| Corrêa (2018b)^27^ | Universidade Federal de Santa Catarina | Sao Paulo Medical Journal | English | Cross-sectional | Quantitative analytical | South | State capitals | Primary | School children, Adolescents | Community | Physical | No |
| Costa (2019)^28^ | Universidade Federal de Minas Gerais | BMC Public Health | English | Ecological | Quantitative descriptive | Southeast | State capitals | Primary | - | Community | Economic, Physical | Glanz et al (2005)^113^ |
| Costa (2015)^29^ | Universidade Federal de Minas Gerais | Cadernos de Saúde Pública | English, Portuguese | Cross-sectional | Quantitative descriptive | Southeast | State capitals | Primary, Secondary | - | Community | Physical | No |
| Cunha (2022)^30^ | Universidade Federal do Rio Grande do Sul | Ciência & Saúde Coletiva | English, Portuguese | Cross-sectional | Quantitative analytical | South | State capitals | Primary | - | Consumer | Sociocultural, Physical | Swinburn et al (2013)^116^ |
| Curioni (2020)^31^ | Universidade do Estado do Rio de Janeiro | Public Health | English | Cross-sectional | Quantitative analytical | Southeast | State capitals | Secondary | Adults | Community | Physical | No |
| Davies (2017)^32^ | Lancaster University | Annals of the American Association of Geographers | English | Ecological | Quantitative analytical | North | State capitals , Metropolitan regions | Primary | - | Community, Consumer | Economic, Sociocultural, Physical | No |
| Domingos (2022)^33^ | Universidade Federal de Viçosa | Ciência & Saúde Coletiva | English | Cross-sectional | Quantitative analytical | Southeast | Countryside | Secondary | Adults, Elderly | Community | Physical | Swinburn et al (1999)^117^ |
| Duran (2015)^34^ | Universidade de São Paulo | Revista de Saúde Pública | English | Studies of validity or reliability of instruments and measures | Quantitative descriptive | Southeast | State capitals | Primary | - | Consumer | Economic, Physical | Glanz et al (2005)^113^ |
| Duran (2013)^35^ | Universidade de São Paulo | Health & Place | English | Cross-sectional | Quantitative analytical | Southeast | State capitals | Primary | - | Community | Physical , Sociocultural | Glanz et al (2005)^113^ |
| Duran (2016)^36^ | Universidade de São Paulo | Public Health Nutrition | English | Cross-sectional | Quantitative analytical | Southeast | State capitals | Primary, Secondary | Adults | Community | Sociocultural, Physical | No |
| Duran (2019)^37^ | Universidade Estadual de Campinas | Nutrients | English | Ecological | Quantitative descriptive | Northeast, Southeast | State capitals | Primary | - | Consumer, Information | Physical | No |
| Estima (2014)^38^ | Universidade de São Paulo | Journal of Nutrition Education and Behavior | English | Cross-sectional | Quantitative descriptive | Southeast | State capitals | Primary | Adolescents | Organizational | Physical | No |
| Franco (2020)^39^ | Faculdade Bezerra de Araújo | Revista de Nutrição | English | Ecological | Quantitative descriptive | Southeast | State capitals | Primary | - | Organizational | Economic, Physical | No |
| Franco (2013)^40^ | Universidade do Estado do Rio de Janeiro | Revista de Saúde Pública | English, Portuguese | Community trial | Quantitative analytical | Southeast | State capitals | Primary | Adults | Organizational | Physical | No |
| Franco (2022)^133^ | Centro Universitário Serra dos Órgãos | Ciência & Saúde Coletiva | English, Portuguese | Studies of validity or reliability of instruments and measures | Quantitative descriptive | Southeast | State capitals | Primary | - | Organizational | Physical | Glanz et al (2005)^113^, Caspi et al (2012)^121^ |
| Freitas (2019)^41^ | Universidade Federal de Minas Gerais | Nutrition | English | Cross-sectional | Quantitative analytical | Southeast | State capitals | Primary | Adults | Consumer | Physical | No |
| Froelich (2021)^42^ | Instituto Federal do Mato Grosso | Revista Chilena de Nutrición | English | Cross-sectional | Quantitative descriptive | North, South, Northeast, Southeast, Central-West | State capitals , Metropolitan regions, Countryside | Secondary | Adolescents | Organizational | Physical | No |
| Giacomelli (2017)^43^ | Universidade Federal de Santa Catarina | Brazilian Journal of Food Technology | Portuguese | Cross-sectional | Quantitative descriptive | South | State capitals | Primary | - | Organizational, Community | Physical , Policy | No |
| Gomes (2022a)^44^ | Universidade Federal de Minas Gerais | Ciência & Saúde Coletiva | English | Cross-sectional | Quantitative analytical | Southeast | State capitals | Secondary | Adults | Community | Physical | No |
| Gomes (2022b)^45^ | Universidade Federal de Minas Gerais | Appetite | English | Cross-sectional | Quantitative analytical | Southeast | State capitals | Secondary | Adults | Community | Physical , Sociocultural | Glanz et al (2005)^113^, Story et al (2008)^118^ |
| Gonçalves (2019)^46^ | Universidade de Brasília | Public Health Nutrition | English | Cross-sectional | Quantitative analytical | North, South, Northeast, Southeast, Central-West | State capitals , Metropolitan regions, Countryside | Secondary | Adolescents | Organizational, Community | Physical | No |
| Grilo (2022)^134^ | Universidade Estadual de Campinas | Ciência & Saúde Coletiva | English, Portuguese | Ecological | Quantitative descriptive | Southeast | Metropolitan regions | Secondary | - | Community | Physical | No |
| Guedes (2011)^47^ | Universidade Estadual de Londrina | Revista Panamericana de Salud Publica | English | Cross-sectional | Quantitative analytical | Southeast | Countryside | Secondary | School children, Adolescents | Organizational | Physical , Sociocultural | No |
| Henriques (2021)^48^ | Universidade Federal Fluminense | Ciência & Saúde Coletiva | English, Portuguese | Cross-sectional | Quantitative descriptive | Southeast | Metropolitan regions | Primary | - | Community | Physical | Swinburn et al (1999)117 |
| Honório (2022)^49^ | Universidade Federal de Minas Gerais | Food Security | English | Cross-sectional | Quantitative descriptive | Southeast | State capitals | Secondary | - | Community | Sociocultural, Physical | No |
| Honório (2021)^50^ | Universidade Federal de Minas Gerais | International Journal for Equity in Health | English | Ecological | Quantitative descriptive | Southeast | State capitals | Secondary | - | Community | Sociocultural, Physical | No |
| Horta (2020)^51^ | Universidade Federal de Minas Gerais | Journal of Nutritional Science | English | Cross-sectional | Quantitative descriptive | Southeast | State capitals | Primary | - | Community | Physical | No |
| Horta (2021a)^52^ | Universidade Federal de Minas Gerais | Public Health Nutrition | English | Cross-sectional | Quantitative descriptive | Southeast | State capitals | Primary | - | Digital | Physical | Granheim et al (2020)^119^ |
| Horta (2021b)^53^ | Universidade Federal de Minas Gerais | Jornal de Pediatria | English | Cross-sectional | Quantitative descriptive | - | - | Primary | - | Information | Physical | No |
| Horta (2021c)^54^ | Universidade Federal de Minas Gerais | British Journal of Nutrition | English | Cross-sectional | Quantitative descriptive | North, South, Northeast, Southeast, Central-West | State capitals | Primary | - | Digital | Physical | Granheim et al (2020) ^119^ |
| Jaime (2006)^55^ | Universidade de São Paulo | Revista Chilena de Nutrición | English | Community trial | Quantitative descriptive | Southeast | State capitals | Primary | Adults | Organizational | Physical | No |
| Jaime (2011)^56^ | Universidade de São Paulo | Journal of Urban Health | English | Cross-sectional | Quantitative analytical | Southeast | State capitals | Secondary | Adults | Community | Sociocultural, Physical | No |
| José (2021)^57^ | Universidade do Estado do Rio de Janeiro | Public Health Nutrition | English | Cross-sectional | Quantitative descriptive | Southeast | State capitals | Primary | - | Organizational | Physical | No |
| Justiniano (2022)^58^ | Universidade Federal de Ouro Preto | Public Health Nutrition | English | Ecological | Quantitative descriptive | Southeast | State capitals | Secondary | - | Community | Sociocultural, Physical | No |
| Leite (2021)^59^ | Universidade de São Paulo | Food Security | English | Ecological | Quantitative descriptive | Southeast | Countryside | Secondary | - | Community | Sociocultural, Physical | No |
| Leite (2019)^60^ | Universidade de São Paulo | Public Health Nutrition | English | Ecological | Quantitative analytical | Southeast | Countryside | Primary, Secondary | - | Consumer | Sociocultural, Physical , Economic | No |
| Leite (2012)^61^ | Universidade de São Paulo | Jornal de Pediatria | English, Portuguese | Ecological | Quantitative descriptive | Southeast | Metropolitan regions | Primary | - | Community | Physical , Sociocultural | Glanz et al (2005)^113^ |
| Leite (2018)^62^ | Universidade Federal de São Paulo | Public Health Nutrition | English | Cross-sectional | Quantitative analytical | Southeast | Metropolitan regions | Primary | Preschool children | Community | Sociocultural, Physical | Glanz et al (2005) ^113^ |
| Leite (2022)^63^ | Universidade de São Paulo | Cadernos de Saúde Pública | English, Portuguese | Cross-sectional | Quantitative analytical | Southeast | State capitals | Primary | Adolescents | Organizational | Physical | FAO (2019)^120^ |
| Leme (2017)^64^ | Universidade de São Paulo | Nutrire | English | Cross-sectional | Quantitative analytical | Southeast | State capitals | Primary | Adolescents mulheres | Organizational | Sociocultural, Physical | No |
| Lopes (2021)^65^ | Universidade Federal de Minas Gerais | Public Health Nutrition | English | Ecological | Quantitative analytical | Southeast | State capitals | Secondary | - | Community | Sociocultural, Physical | No |
| Lopes (2017)^66^ | Universidade Federal de Minas Gerais | Saúde e Sociedade | English, Portuguese | Commentary or Opinion | - | Southeast | State capitals | - | - | Consumer | Physical , Policy | No |
| Lopes (2022)^135^ | Universidade Federal de Minas Gerais | Ciência & Saúde Coletiva | English, Portuguese | Ecological | Quantitative descriptive | Southeast | State capitals | Secondary | - | Community | Physical | No |
| Martins (2013)^67^ | Universidade Federal de São Paulo | Journal of Nutrition Education and Behavior | English | Studies of validity or reliability of instruments and measures | Quantitative descriptive | Southeast | Metropolitan regions | Primary | - | Community | Physical | No |
| Matozinhos (2015)^68^ | Universidade Federal de Minas Gerais | Preventive Medicine Reports | English | Cross-sectional | Quantitative analytical | Southeast | State capitals | Secondary | Adults | Community | Physical | No |
| Maziero (2017)^69^ | Universidade de São Paulo | Revista Brasileira de Epidemiologia | English, Portuguese | Cross-sectional | Quantitative descriptive | Southeast | State capitals | Primary | Adults | Consumer | Sociocultural, Physical | No |
| Mendes (2021)^70^ | Universidade Federal de Minas Gerais | Cadernos de Saúde Pública | English, Portuguese | Commentary or Opinion | - | - | - | - | - | - | Policy | No |
| Mendes (2022)^71^ | Universidade Federal de Minas Gerais | Public Health Nutrition | English | Commentary or Opinion | - | - | - | - | - | Organizational, Community, Information, Digital | Economic, Sociocultural, Physical | No |
| Mendes (2013)^72^ | Universidade Federal de Juiz de Fora | BMC Public Health | English | Cross-sectional | Quantitative analytical | Southeast | State capitals | Secondary | Adults | Community | Sociocultural, Physical | No |
| Mendonça (2019)^73^ | Universidade Federal de Minas Gerais | Revista de Saúde Pública | English, Portuguese | Cross-sectional | Quantitative analytical | Southeast | State capitals | Primary | Adults | Community | Physical | No |
| Menezes (2018a)^74^ | Universidade Federal de Minas Gerais | Public Health Nutrition | English | Cross-sectional | Quantitative analytical | Southeast | State capitals | Primary | Adults | Community | Physical , Economic | Glanz et al (2005)^113^ |
| Menezes (2018b)^77^ | Universidade Federal de Minas Gerais | Appetite | English | Cross-sectional | Quantitative analytical | Southeast | State capitals | Primary | Adults | Community | Sociocultural, Physical | No |
| Menezes (2016)^75^ | Universidade Federal de Minas Gerais | Preventive Medicine Reports | English | Cross-sectional | Quantitative descriptive | Southeast | State capitals | Primary | Adults | Community | Physical , Sociocultural | No |
| Menezes (2021)^76^ | Escola Nacional de Saúde Pública Sérgio Arouca | Journal of Urban Health | English | Studies of validity or reliability of instruments and measures | Quantitative descriptive | Southeast | State capitals | Primary, Secondary | - | Community | Physical , Sociocultural | No |
| Menezes (2022)^131^ | Universidade Federal de Alagoas | Health and Place | English | Cross-sectional | Quantitative analytical | Northeast | State capitals, Metropolitan regions | Primary | Adults | Community, Consumer | Physical | Glanz et al (2005)^113^ |
| Monteiro (2022)^122^ | Universidade Federal de Santa Catarina | Ciência & Saúde Coletiva | English | Cross-sectional | Quantitative analytical | South | Metropolitan regions | Primary | Adults | Community | Physical | No |
| Moreira (2020)^78^ | Universidade Federal do Tocantins | BMC Pediatrics | English | Cross-sectional | Quantitative analytical | North | State capitals | Primary | School children | Organizational | Physical | No |
| Nogueira (2018)^79^ | Universidade de São Paulo | International Journal of Environmental Research and Public Health | English | Cross-sectional | Quantitative analytical | Southeast | State capitals | Secondary | Adolescents | Consumer | Sociocultural, Physical | No |
| Nogueira (2020)^80^ | Universidade de São Paulo | Cadernos de Saúde Pública | English | Cross-sectional | Quantitative analytical | Southeast | State capitals | Secondary | Adolescents | Community | Physical | No |
| Novaes (2022)^136^ | Universidade Federal de Viçosa | Ciência & Saúde Coletiva | English | Ecological | Quantitative descriptive | Southeast | Countryside | Primary | - | Organizational | Physical | No |
| Oliveira (2021)^81^ | Universidade Estadual de Santa Cruz | Finisterra | Portuguese | Ecological | Quantitative descriptive | Northeast | Countryside | Secondary | - | Community | Sociocultural, Physical | No |
| Oliveira (2022)^139^ | Universidade Federal de Pernambuco | Food Policy | English | Cross-sectional | Quantitative analytical | Northeast | State capitals, Metropolitan regions | Primary | Adults | Community, Consumer | Physical | No |
| Paulitsch (2021)^82^ | Universidade Estadual do Rio Grande do Sul | Preventive Medicine Reports | English | Cross-sectional | Quantitative analytical | South | Countryside | Primary, Secondary | Adults | Community | Physical | No |
| Pereira (2021)^83^ | Universidade Federal de Juiz de Fora | Revista de Nutrição | English | Ecological | Quantitative descriptive | Southeast | Countryside | Primary | - | Consumer | Physical , Economic | No |
| Pereira (2019)^84^ | Instituto Federal de Minas Gerais | British Food Journal | English | Cross-sectional | Quantitative descriptive | - | - | Primary | - | Digital | Physical | No |
| Pereira (2022)^85^ | Instituto Federal de Minas Gerais | Journal of Food Science and Technology | English | Ecological | Quantitative descriptive | Southeast | - | Primary | - | Information | Physical | No |
| Peres (2021)^86^ | Universidade Federal de Minas Gerais | Cadernos de Saúde Pública | Portuguese | Ecological | Quantitative descriptive | Southeast | State capitals | Primary | - | Organizational, Community | Sociocultural, Physical | Glanz et al (2005)^113^, FAO (2019)^120^ |
| Pessoa (2015)^87^ | Universidade Federal de Minas Gerais | BMC Public Health | English | Cross-sectional | Quantitative analytical | Southeast | State capitals | Secondary | Adults | Community | Sociocultural, Physical | No |
| Pessoa (2014)^88^ | Universidade Federal de Viçosa | Nutrición Hopitalaria | English | Cross-sectional | Quantitative descriptive | Southeast | State capitals | Secondary | Adults | Community | Sociocultural, Physical | No |
| Prado (2022)^137^ | Universidade Federal de Santa Catarina | Revista de Nutrição | English | Cross-sectional | Qualitative | South | State capitals | Primary | Adults | Community | Physical | No |
| Pulz (2017)^89^ | Universidade Federal de Santa Catarina | Perspectives in Public Health | English | Ecological | Quantitative descriptive | South | State capitals | Primary | - | Organizational | Physical | Glanz et al (2005)^113^ |
| Rocha (2021a)^90^ | Universidade Federal de Minas Gerais | Journal of the Academy of Nutrition and Dietetics | English | Cross-sectional | Quantitative analytical | North, South, Northeast, Southeast, Central-West | State capitals , Metropolitan regions, Countryside | Secondary | Adolescents | Organizational | Physical | No |
| Rocha (2021b)^91^ | Universidade Federal de Minas Gerais | Food, Culture & Society | English | Ecological | Quantitative descriptive | Southeast | State capitals | Secondary | - | Community | Physical , Sociocultural | No |
| Rocha (2021c)^92^ | Universidade Federal de Minas Gerais | Frontiers in Nutrition | English | Cross-sectional | Quantitative analytical | North, South, Northeast, Southeast, Central-West | State capitals , Metropolitan regions, Countryside | Secondary | Adolescents | Organizational, Community | Physical | No |
| Rodrigues (2021)^93^ | Universidade Federal de Minas Gerais | Public Health Nutrition | English | Commentary or Opinion | - | - | - | - | - | Information | Physical , Policy | No |
| Rossi (2019)^94^ | Universidade Federal de Santa Catarina | Journal of Public Health | English | Cross-sectional | Quantitative analytical | South | State capitals | Secondary | School children | Organizational | Sociocultural, Physical | No |
| Sanches (2018)^95^ | Universidade Federal de São Paulo | Geografares | Portuguese | Studies of validity or reliability of instruments and measures | Quantitative descriptive | Southeast | Metropolitan regions | Primary | - | Community | Physical | No |
| Santos (2013)^96^ | Escola Nacional de Saúde Pública Sérgio Arouca | Revista de Saúde Pública | English, Portuguese | Studies of validity or reliability of instruments and measures | Quantitative descriptive | South, Northeast, Southeast | State capitals | Primary | Adults, Elderly | Community | Physical | No |
| Santos (2021)^97^ | Universidade de São Paulo | Phisys | Portuguese | Cross-sectional | Qualitative | North | Countryside | Primary | Adults | Consumer | Economic, Sociocultural, Physical | No |
| Scaciota (2022)^98^ | Universidade de São Paulo | Journal of Public Health | English | Studies of validity or reliability of instruments and measures | Quantitative descriptive | Southeast | State capitals | - | - | Consumer | Physical | Glanz et al (2005)^113^ |
| Serafim (2022)^138^ | Universidade de São Paulo | Frontiers in Nutrition | English | Cross-sectional | Quantitative descriptive | Southeast | Metropolitan regions | Primary | - | Consumer | Physical | No |
| Silva (2019)^99^ | Universidade Federal de Viçosa | Cadernos de Saúde Pública | Portuguese | Cross-sectional | Quantitative analytical | Southeast | Countryside | Primary, Secondary | Adults | Community | Sociocultural, Physical | Swinburn et al (1999)^117^ |
| Silva (2021a)^100^ | Universidade Federal de Minas Gerais | PLoS One | English | Cross-sectional | Quantitative analytical | North, South, Northeast, Southeast, Central-West | State capitals , Metropolitan regions, Countryside | Secondary | Adolescents | Organizational | Physical , Sociocultural | No |
| Silva (2021b)^102^ | Universidade Federal do Paraná | Ciência & Saúde Coletiva | Portuguese | Ecological | Quantitative analytical | South | State capitals | Primary | - | Community | Sociocultural, Physical | No |
| Silva (2022)^101^ | Universidade Federal de Minas Gerais | BMC Public Health | English | Cross-sectional | Quantitative analytical | Southeast | State capitals | Secondary | Pregnant and/or lactating women | Community | Physical | No |
| Silva (2022)^127^ | Universidade Federal de Viçosa | Health Promotion International | English | Cross-sectional | Quantitative analytical | Southeast | Countryside | Primary | Adults | Community, Consumer | Physical | No |
| Soares (2014)^103^ | Universidade Federal de Pelotas | Revista de Nutrição | English | Cross-sectional | Quantitative descriptive | South | Metropolitan regions | Primary | Adults | Organizational | Sociocultural, Physical | No |
| Souza (2022)^104^ | Universidade de São Paulo | Revista de Saúde Pública | English, Portuguese | Cross-sectional | Quantitative analytical | North, South, Northeast, Southeast, Central-West | State capitals , Metropolitan regions, Countryside | Secondary | School children | Organizational, Community | Physical | No |
| Souza (2022)^132^ | Universidade Federal de Santa Catarina | Frontiers in Aging | English | Cross-sectional | Quantitative analytical | South | State capitals | Primary, Secondary | Elderly | Community | Physical | No |
| Tavares (2021)^105^ | Universidade do Estado do Rio de Janeiro | Foods | English | Studies of validity or reliability of instruments and measures | Quantitative descriptive | Southeast | State capitals | Secondary | - | Organizational | Physical | Glanz et al (2005)^113^, Caspi et al (2012)^121^ |
| Vale (2021)^106^ | Universidade Federal do Rio Grande do Norte | Ciência & Saúde Coletiva | Portuguese | Cross-sectional | Quantitative analytical | North, South, Northeast, Southeast, Central-West | State capitals , Metropolitan regions, Countryside | Primary | Adolescents | Organizational | Physical | No |
| Velásquez-Meléndez (2013)^107^ | Universidade Federal de Minas Gerais | Cadernos de Saúde Pública | English | Cross-sectional | Quantitative analytical | Southeast | State capitals | Secondary | Adults | Community | Sociocultural, Physical | Swinburn et al (1999)^117^ |
| Vepsäläinen (2015)^108^ | Universidade de Helsinque | International Journal of Obesity Supplements | English | Cross-sectional | Quantitative analytical | Southeast | Metropolitan regions | Secondary | School children | Organizational | Physical | Glanz et al (2005)^113^ |
| Willets (2019)^109^ | Universidade de São Paulo | Global Heart | English | Cross-sectional | Quantitative analytical | South, Northeast, Southeast | State capitals | Secondary | Adults, Elderly | Community | Physical | No |
| Wognski (2019)^110^ | Universidade Positivo | Brazilian Journal of Food Technology | Portuguese | Cross-sectional | Quantitative descriptive | South | State capitals | Primary | - | Organizational | Physical , Policy | No |
| Zuccolotto (2013)^111^ | Universidade de São Paulo | Revista de Nutrição | Portuguese | Studies of validity or reliability of instruments and measures | Quantitative descriptive | Southeast | Countryside | Primary | Pregnant and/or lactating women | Community | Physical | No |
| Zuccolotto (2015)^112^ | Universidade de São Paulo | Archivos LatinoAmericanos de Nutrición | English | Cross-sectional | Quantitative descriptive | Southeast | Metropolitan regions | Primary | Pregnant and/or lactating women | Community | Physical | No |

1. Almeida LFF, Novaes TG, Pessoa MC et al. (2021a) Fruit and vegetable consumption among older adults: influence of urban food environment in a medium-sized Brazilian city. *Public Health Nutr* 24, 15, 4878-4887. doi: 10.1017/S136898002000467X.

2. Almeida LFF, Novaes TG, Pessoa MC et al. (2021b) Socioeconomic Disparities in the Community Food Environment of a Medium-Sized City of Brazil. *J Am Coll Nutr* 40, 3, 253-260. doi: 10.1080/07315724.2020.1755911.

3. Almeida L, Scagliusi F, Duran A et al. (2018). Barriers to and facilitators of ultra-processed food consumption: Perceptions of Brazilian adults. *Public Health Nutr* *21,* 1, 68-76. doi:10.1017/S1368980017001665.

4. Alves MA, Pinho MGM, Corrêa EM et al. (2019) Parental Perceived Travel Time to and Reported Use of Food Retailers in Association with School Children's Dietary Patterns. *Int J Environ Res Public Health* 16, 5, 824. doi: 10.3390/ijerph16050824.

5. Andretti B, Goldszmidt RB, Andrade EB (2021) How changes in menu quality associate with subsequent expenditure on (un)healthy foods and beverages in school cafeterias: A three-year longitudinal study. *Prev Med* 146,106456. doi: 10.1016/j.ypmed.2021.106456.

6. Araújo ML, Silva GB, Rocha LL et al. (2022) Characteristics of the community food environment and home surroundings of households beneficiaries of the "Bolsa Família" (family aid) program. *Cien Saude Colet* 27, 2, 641-651. doi: 10.1590/1413-81232022272.38562020.

7. Assis MM, Leite MA, Côrtes AJ et al. (2018) Overweight, perceived environment, and social deprivation: a study on the perception of parents or guardians. *Rev Paul Pediatr* 36, 4, 466-473. doi: 10.1590/1984-0462/;2018;36;4;00011.

8. Assis MM, Leite MA, Carmo ASD et al. (2019) Food environment, social deprivation and obesity among students from Brazilian public schools. *Public Health Nutr* 22, 11, 1920-1927. doi: 10.1017/S136898001800112X.

9. Azeredo CM, de Rezende LF, Canella DS et al. (2016) Food environments in schools and in the immediate vicinity are associated with unhealthy food consumption among Brazilian adolescents. *Prev Med* 88, 73-9. doi: 10.1016/j.ypmed.2016.03.026.

10. Azeredo CM, Leite MA, Rauber F et al. (2020) Are laws restricting soft drinks sales in Brazilian schools able to lower their availability? *Rev Saúde Pública* 54. doi: 10.11606/s1518-8787.2020054001227.

11. Backes V, Bairros F, Cafruni CB et al. (2019) Food environment, income and obesity: a multilevel analysis of a reality of women in Southern Brazil. *Cad Saúde Pública* 35, 8. doi: 10.1590/0102-311X00144618.

12. Backes V, Costa JSD, Bairros FS et al. (2021) Food environment in São Leopoldo, Rio Grande do Sul, Brazil: the association with neighborhood sociodemographic variables. *Ciênc saúde coletiva* 26, 5. doi: 10.1590/1413-81232021265.15972019.

13. Bandoni DH, Canella DS (2021) Can Eating Food Offered by Schools Have a Positive Influence on Nutritional Status of Children? An Example from Brazil. *Health Behav Policy Rev* 8, 3, 202-211. doi: 10.14485/HBPR.8.3.2.

14. Bandoni DH, Sarno F, Jaime PC (2011) Impact of an intervention on the availability and consumption of fruits and vegetables in the workplace. *Public Health Nutr* 14, 6, 975-81. doi: 10.1017/S1368980010003460.

15. Bandoni DH, Bombem KCM, Marchioni DML et al. (2010) The influence of the availability of fruits and vegetables in the workplace on the consumption of workers. *Nutr Food Sci* 40, 1, 20-25. doi: 10.1108/00346651011015872.

16. Barbosa R, Henriques P, Guerra H et al. (2020) Food environment of a Brazilian public university: challenges to promote healthy eating. *Rev chi. nutr* 47, 3, 443-448. doi: 10.4067/S0717-75182020000300443.

17. Borges CA, Jaime PC (2019) Development and evaluation of food environment audit instrument: AUDITNOVA. *Rev Saúde Pública* 53. doi: 10.11606/s1518-8787.2019053001316.

18. Borges CA, Gabe KT, Canella DS et al. (2022) Characterization of barriers and facilitators for adequate and healthy eating in the consumer's food environment. *Cad Saude Publica* 37, 1, e00157020. doi: 10.1590/0102-311X00157020.

19. Borges CA, Cabral-Miranda W, Jaime PC (2018) Urban Food Sources and the Challenges of Food Availability According to the Brazilian Dietary Guidelines Recommendations. *Sustainability* 10, 12, 4643. doi: 10.3390/su10124643.

20. Borges CA, Gabe KT, Jaime PC (2021) Consumer Food Environment Healthiness Score: Development, Validation, and Testing between Different Types of Food Retailers. *Int J Environ Res Public Health* 18, 7, 3690. doi: 10.3390/ijerph18073690.

21. Botelho LV, Cardoso LO, Canella DS (2020) COVID-19 and the digital food environment in Brazil: reflections on the pandemic's influence on the use of food delivery apps. *Cad Saude Publica* 23, 36(11), e00148020. doi: 10.1590/0102-311X00148020.

22. Camargo DFM, Belon AP, Marín-León L et al. (2019) Comparing food environment and food purchase in areas with low and high prevalence of obesity: data from a mapping, in-store audit, and population-based survey. *Cad Saúde Pública* 35, 9. doi: 10.1590/0102-311X00247218.

23. Carmo ASD, Assis MM, Cunha CF et al. (2018) The food environment of Brazilian public and private schools. *Cad Saude Publica* 34, 12, e00014918. doi: 10.1590/0102-311X00014918.

24. Castro IRR, Canella DS (2022) Organizational Food Environments: Advancing Their Conceptual Model. *Foods* 11, 7, 993. doi: 10.3390/foods11070993.

25. Chor D, Cardoso LO, Nobre AA et al. (2016) Association between perceived neighbourhood characteristics, physical activity and diet quality: results of the Brazilian Longitudinal Study of Adult Health (ELSA-Brasil). *BMC Public Health* 16, 751. doi: 10.1186/s12889-016-3447-5.

26. Corrêa EN, Rossi CE, das Neves J et al. (2018a) Utilization and environmental availability of food outlets and overweight/obesity among schoolchildren in a city in the south of Brazil. *J Public Health* 40, 1, 106-113. doi: 10.1093/pubmed/fdx017.

27. Corrêa EN, Retondario A, Alves MA et al. (2018b) Utilization of food outlets and intake of minimally processed and ultra-processed foods among 7 to 14-year-old schoolchildren. A cross-sectional study. *Sao Paulo Med J* 136, 03. doi: 10.1590/1516-3180.2017.0211061217.

28. Costa BVL, Menezes MC, Oliveira CDL et al. (2019) Does access to healthy food vary according to socioeconomic status and to food store type? an ecologic study. *BMC Public Health* 19, 1, 775. doi: 10.1186/s12889-019-6975-y.

29. Costa BVL, Oliveira CL, Lopes ACS (2015) Food environment of fruits and vegetables in the territory of the Health Academy Program. *Cad Saúde Pública* 31, 1. doi: 10.1590/0102-311X00027114.

30. Cunha CML, Canuto R, Rosa PBZ et al. (2022) Association between dietary patterns and socioeconomic factors and food environment in a city in the South of Brazil. *Cien Saude Colet* 27, 2, 687-700. doi: 10.1590/1413-81232022272.37322020.

31. Curioni CC, Boclin KLS, Silveira IH et al. (2020) Neighborhood food environment and consumption of fruit and leafy vegetables: Pro-Saude Study, Brazil. *Public Health* 182, 7-12. doi: 10.1016/j.puhe.2020.01.004.

32. Davies G, Frausin G, Parry L (2017) Are There Food Deserts in Rainforest Cities?, *Ann Assoc Am Geogr* 107, 4, 794-811, doi: 10.1080/24694452.2016.1271307.

33. Domingos ALG, Hermsdorff HHM, Mendes LL et al. (2022) Built and social environments and overweight among Brazilian adults from medium-sized city: CUME Project. *Cien Saude Colet* 27, 2, 771-782. doi: 10.1590/1413-81232022272.02422021.

34. Duran AC, Lock K, Latorre MR et al. (2015) Evaluating the use of in-store measures in retail food stores and restaurants in Brazil. *Rev Saude Publica* 49, 80. doi: 10.1590/S0034-8910.2015049005420.

35. Duran AC, Diez Roux AV, Latorre MR et al. (2013) Neighborhood socioeconomic characteristics and differences in the availability of healthy food stores and restaurants in Sao Paulo, Brazil. *Health Place* 23, 39-47. doi: 10.1016/j.healthplace.2013.05.001.

36. Duran AC, de Almeida SL, Latorre MR et al. (2016) The role of the local retail food environment in fruit, vegetable and sugar-sweetened beverage consumption in Brazil. *Public Health Nutr* 19, 6, 1093-102. doi: 10.1017/S1368980015001524.

37. Duran AC, Ricardo CZ, Mais LA et al. (2019) Conflicting Messages on Food and Beverage Packages: Front-of-Package Nutritional Labeling, Health and Nutrition Claims in Brazil. *Nutrients* 11, 12, 2967. doi: 10.3390/nu11122967.

38. Estima CC, Bruening M, Hannan PJ et al. (2014) A cross-cultural comparison of eating behaviors and home food environmental factors in adolescents from São Paulo (Brazil) and Saint Paul-Minneapolis (US). *J Nutr Educ Behav* 46, 5, 370-5. doi: 10.1016/j.jneb.2014.01.007.

39. Franco AS, Canella DS, Perez PMP et al. (2020) University food environment: characterization and changes from 2011 to 2016 in a Brazilian public university. *Rev Nutr* 33. doi: 10.1590/1678-9865202033e200058.

40. Franco AS, Castro IR, Wolkoff DB (2013) Impact of the promotion of fruit and vegetables on their consumption in the workplace. *Rev Saude Publica* 47, 1, 29-36. doi: 10.1590/s0034-89102013000100005.

41. Freitas PP, de Menezes MC, Lopes ACS (2019) Consumer food environment and overweight. *Nutrition* 66, 108-114. doi: 10.1016/j.nut.2019.04.013.

42. Froelich M, Souza BSN, Andrade ACS et al. (2021) School environment and adherence to school meals among Brazilian public-school adolescents. *Rev chil nutr* 48, 5, 732-740. doi: 10.4067/S0717-75182021000500732.

43. Giacomelli SC, Londero AM, Benedetti FJ et al. (2017) Informal and formal food trade in the school environment in a city in the central region of Rio Grande do Sul, Brazil. *Braz J Food Technol* 20. doi: 10.1590/1981-6723.13616.

44. Gomes CS, Mendes LL, Vieira MA et al. (2022a) Spatial distribution of sedentary behavior and unhealthy eating habits in Belo Horizonte, Brazil: the role of the neighborhood environment. *Cien Saude Colet* 27, 4, 1503-1512. doi: 10.1590/1413-81232022274.47232020.

45. Gomes CS, Silveira EA, Velasquez-Melendez G (2022b) Neighborhood environment is associated with unhealthy food intake in a Brazilian urban area. *Appetite* 172, 105972. doi: 10.1016/j.appet.2022.105972.

46. Gonçalves VS, Duarte EC, Dutra ES et al. (2019) Characteristics of the school food environment associated with hypertension and obesity in Brazilian adolescents: a multilevel analysis of the Study of Cardiovascular Risks in Adolescents (ERICA). *Public Health Nutr* 22, 14, 2625-2634. doi: 10.1017/S1368980019001010.

47. Guedes DP, Rocha GD, Silva AJ et al. (2011) Effects of social and environmental determinants on overweight and obesity among Brazilian schoolchildren from a developing region. *Rev Panam Salud Publica* 30,4, 295-302.

48. Henriques P, Alvarenga CRT, Ferreira DM et al. (2021) Food environment surrounding public and private schools: an opportunity or challenge for healthy eating? *Cien Saude Colet* 26, 8, 3135-3145. doi: 10.1590/1413-81232021268.04672020.

49. Honório OS, Horta PM, Pessoa MC et al. (2022) Food deserts and food swamps in a Brazilian metropolis: comparison of methods to evaluate the community food environment in Belo Horizonte. *Food Sec* 14, 695–707. doi: 10.1007/s12571-021-01237-w.

50. Honório OS, Pessoa MC, Gratão LHA et al. (2021) Social inequalities in the surrounding areas of food deserts and food swamps in a Brazilian metropolis. *Int J Equity Health* 20, 1, 168. doi: 10.1186/s12939-021-01501-7.

51. Horta PM, Souza JPM, Freitas PP et al. (2020) Food availability and advertising within food outlets around primary healthcare services in Brazil. *J Nutr Sci* 11, 9, e49. doi: 10.1017/jns.2020.45.

52. Horta PM, Souza JPM, Rocha LL et al. (2021a) Digital food environment of a Brazilian metropolis: food availability and marketing strategies used by delivery apps. *Public Health Nutr* 24, 3, 544-548. doi: 10.1017/S1368980020003171.

53. Horta PM, Machado BB, Souza LV (2021b) Food content on children movies from 2013 to 2018: taking food processing into account. *J Pediatr* 97, 3. doi: 10.1016/j.jped.2020.06.006.

54. Horta P, Matos J, Mendes L (2021c) Digital food environment during the coronavirus disease 2019 (COVID-19) pandemic in Brazil: An analysis of food advertising in an online food delivery platform. *Br J Nutr* *126,* 5, 767-772. doi: 10.1017/S0007114520004560.

55. Jaime PC, Machado FMS, Westphal MF et al. (2006) Impact of a community-based intervention to increase fruit and vegetable consumption among low-income families from Sao Paulo, Brasil. *Rev chil nutr* 33, 1, 266-271. doi: 10.4067/S0717-75182006000300008.

56. Jaime PC, Duran AC, Sarti FM et al. (2011) Investigating environmental determinants of diet, physical activity, and overweight among adults in Sao Paulo, Brazil. *J Urban Health* 88, 3, 567-81. doi: 10.1007/s11524-010-9537-2.

57. José MER, Castro IRR, Canella DS (2021) Evaluation of the food environment of public hospitals in a Brazilian metropolis. *Public Health Nutr* 24, 18, 6477-6487. doi: 10.1017/S1368980021003992.

58. Justiniano ICS, de Menezes MC, Mendes LL et al. (2022) Retail food environment in a Brazilian metropolis over the course of a decade: evidence of restricted availability of healthy foods. *Public Health Nutr* 25, 9, 2584-2592. doi: 10.1017/S1368980022000787.

59. Leite MA, de Assis MM, do Carmo AS et al. (2021) Inequities in the urban food environment of a Brazilian city. *Food Sec* 13, 539–549. doi: 10.1007/s12571-020-01116-w.

60. Leite MA, Assis MM, Carmo ASD et al. (2019) Is neighbourhood social deprivation in a Brazilian city associated with the availability, variety, quality and price of food in supermarkets? *Public Health Nutr* 22, 18, 3395-3404. doi: 10.1017/S1368980019002386.

61. Leite FH, Oliveira MA, Cremm EC et al. (2012) Availability of processed foods in the perimeter of public schools in urban areas. *J Pediatr* 88, 4, 328-34. doi: 10.2223/JPED.2210.

62. Leite FHM, de Carvalho Cremm E, de Abreu DSC et al. (2018) Association of neighbourhood food availability with the consumption of processed and ultra-processed food products by children in a city of Brazil: a multilevel analysis. *Public Health Nutr* 21,1,189-200. doi: 10.1017/S136898001600361X.

63. Leite MA, Azeredo CM, Peres MFT et al. (2022) Availability and consumption of ultra-processed foods in schools in the municipality of São Paulo, Brazil: results of the SP-Proso. *Cad Saude Publica* 37, 1, e00162920. doi: 10.1590/0102-311X00162920.

64. Leme AB, Philippi ST (2017) Home food availability, parents’/caregivers’ support, and family meals influence on dietary servings of low-income urban adolescent girls from Brazil. *Nutrire* 42, 30. doi: 10.1186/s41110-017-0053-y.

65. Lopes MS, Caiaffa WT, Andrade ACS et al. (2021) Spatial inequalities of retail food stores may determine availability of healthful food choices in a Brazilian metropolis. *Public Health Nutr* 1-12. doi: 10.1017/S1368980021002706.

66. Lopes ACS, Menezes MC, Araújo ML (2017) O ambiente alimentar e o acesso a frutas e hortaliças: “Uma metrópole em perspectiva”. *Saude soc* 26, 3, 764–73.

67. Martins PA, Cremm EC, Leite FH et al. (2013) Validation of an adapted version of the nutrition environment measurement tool for stores (NEMS-S) in an urban area of Brazil. *J Nutr Educ Behav* 45, 6, 785-92. doi: 10.1016/j.jneb.2013.02.010.

68. Matozinhos FP, Gomes CS, Andrade AC et al. (2015) Neighbourhood environments and obesity among adults: A multilevel analysis of an urban Brazilian context. *Prev Med Rep* 2, 337-41. doi: 10.1016/j.pmedr.2015.04.019.

69. Maziero CCS, Jaime PC, Duran AC (2017) The influence of meal and food markets in fruit and vegetable consumption among adults in the city of São Paulo. *Rev Bras Epidemiol* 20, 4, 611-623. doi: 10.1590/1980-5497201700040005.

70. Mendes LL, Cardoso LO, Menezes MC et al. (2021) The incorporation of food environments into the Brazilian National Food and Nutrition Policy: an approach to possibilities, advances and challenges. *Cad Saude Publica* 37, 1, e00038621. doi: 10.1590/0102-311X00038621.

71. Mendes LL, Canella DS, Araújo ML et al. (2022) Food environments and the COVID-19 pandemic in Brazil: analysis of changes observed in 2020. *Public Health Nutr* 25, 1, 32-35. doi: 10.1017/S1368980021003542.

72. Mendes LL, Nogueira H, Padez C et al. (2013) Individual and environmental factors associated for overweight in urban population of Brazil. *BMC Public Health* 13, 988. doi: 10.1186/1471-2458-13-988.

73. Mendonça RD, Lopes MS, Freitas PP et al. (2019) Monotony in the consumption of fruits and vegetables and food environment characteristics. *Rev Saúde Pública* 53. doi: 10.11606/S1518-8787.2019053000705.

74. Menezes MC, Diez Roux AV, Costa BVL et al. (2018a) Individual and food environmental factors: association with diet. *Public Health Nutr* 21, 15, 2782-2792. doi: 10.1017/S1368980018001623.

75. Menezes MC, Costa BV, Oliveira CD et al. (2016) Local food environment and fruit and vegetable consumption: An ecological study. *Prev Med Rep* 5, 13-20. doi: 10.1016/j.pmedr.2016.10.015.

76. Menezes MC, de Matos VP, de Pina M et al. (2021) Web Data Mining: Validity of Data from Google Earth for Food Retail Evaluation. *J Urban Health* 98, 285–295. doi: 10.1007/s11524-020-00495-x.

77. Menezes MC, Diez Roux AV, Souza Lopes AC (2018b) Fruit and vegetable intake: Influence of perceived food environment and self-efficacy. *Appetite* 127, 249-256. doi: 10.1016/j.appet.2018.05.011.

78. Moreira RAM, Moreira TR, Costa GD et al. (2020) Multilevel analysis of factors that influence overweight in children: research in schools enrolled in northern Brazil School Health Program. *BMC Pediatr* 20, 1, 188. doi: 10.1186/s12887-020-02096-8.

79. Nogueira LR, Fontanelli MM, Aguiar BS et al. (2018) Access to Street Markets and Consumption of Fruits and Vegetables by Adolescents Living in São Paulo, Brazil. *Int J Environ Res Public Health* 15, 3, 517. doi: 10.3390/ijerph15030517.

80. Nogueira LR, Fontanelli MM, Aguiar BS et al. (2020) Is the local food environment associated with excess body weight in adolescents in São Paulo, Brazil? *Cad Saude Publica* 36, 2, e00048619. doi: 10.1590/0102-311X00048619.

81. Oliveira ES, Jesus AP, Martinez RA (2021) Availability and access to healthy foods in the municipalities of the Ilhéus-Itabuna Microrregion in the State of Bahia (Brazil). *Finisterra: Revista Portuguesa de Geografia* 56, 118, 111-129.

82. Paulitsch RG, Dumith SC (2021) Is food environment associated with body mass index, overweight and obesity? A study with adults and elderly subjects from southern Brazil. *Prev Med Rep* 21, 101313. doi: 10.1016/j.pmedr.2021.101313.

83. Pereira PML, Pereira PF, Castellões ML et al. (2021) Availability and access to food in supermarkets before and during the Covid-19 pandemic in a mid-size city. *Rev Nutr* 34. doi: 10.1590/1678-9865202134e210006.

84. Pereira RC, Angelis-Pereira MC, Carneiro JDS (2019) Exploring claims and marketing techniques in Brazilian food labels. *Br Food J* 121, 7, 1550-1564. doi: 10.1108/BFJ-08-2018-0516.

85. Pereira RC, Souza Carneiro JD, de Angelis Pereira MC (2022) Evaluating nutrition quality of packaged foods carrying claims and marketing techniques in Brazil using four nutrient profile models. *J Food Sci Technol* 59, 4, 1520-1528. doi: 10.1007/s13197-021-05162-w.

86. Peres CMDC, Costa BVL, Pessoa MC et al. (2021) Community food environment and presence of food swamps around schools in a Brazilian metropolis. *Cad Saude Publica* 37, 5, e00205120. doi: 10.1590/0102-311X00205120. Erratum in: *Cad Saude Publica* 37,9, eER205120.

87. Pessoa MC, Mendes LL, Gomes CS et al. (2015) Food environment and fruit and vegetable intake in a urban population: a multilevel analysis. *BMC Public Health* 15, 1012. doi: 10.1186/s12889-015-2277-1.

88. Pessoa MC, Loures Mendes L, Teixeira Caiaffa W et al. (2014) Availability of food stores and consumption of fruit, legumes and vegetables in a Brazilian urban area. *Nutr Hosp* 31, 3, 1438-43. doi: 10.3305/nh.2015.31.3.8245.

89. Pulz IS, Martins PA, Feldman C et al. (2017) Are campus food environments healthy? A novel perspective for qualitatively evaluating the nutritional quality of food sold at foodservice facilities at a Brazilian university. *Perspect Public Health* 137, 2, 122-135. doi: 10.1177/1757913916636414.

90. Rocha LL, Gratão LHA, Carmo ASD et al. (2021a) School Type, Eating Habits, and Screen Time are Associated With Ultra-Processed Food Consumption Among Brazilian Adolescents. *J Acad Nutr Diet* 121, 6, 1136-1142. doi: 10.1016/j.jand.2020.12.010.

91. Rocha LL, Carmo ASD, Jardim MZ et al. (2021b) The community food environment of a Brazilian metropolis. *Food Cult Soc.* doi: 10.1080/15528014.2021.1987027.

92. Rocha LL, Pessoa MC, Gratão LHA et al. (2021c) Characteristics of the School Food Environment Affect the Consumption of Sugar-Sweetened Beverages Among Adolescents. *Front Nutr* 8, 742744. doi: 10.3389/fnut.2021.742744.

93. Rodrigues MB, Matos JP, Horta PM (2021) The COVID-19 pandemic and its implications for the food information environment in Brazil. *Public Health Nutr* 24, 2, 321-326. doi:10.1017/S1368980020004747.

94. Rossi CE, Patrícia de Fragas H, Corrêa EM et al. (2019) Association between food, physical activity, and social assistance environments and the body mass index of schoolchildren from different socioeconomic strata. *J Public Health* 41, 1, e25-e34. doi: 10.1093/pubmed/fdy086.

95. Sanches LD, Lopes RF, Melzer MRTF et al. (2018) Development and validity of an instrument to evaluate the impact of an intervention in food stores at an urban food desert. *Geografares* 25, 396–411. doi: 10.7147/GEO25.17604.

96. Santos SM, Griep RH, Cardoso LO et al. (2013) Cross-cultural adaptation and reliability of measurements on self-reported neighborhood characteristics in ELSA-Brasil. *Rev Saude Publica* 47, 2, 122-30. doi: 10.1590/s0034-8910.2013047003871.

97. Santos CP, Ulian MD, Scagliusi FB et al. (2021) Intertwined between the dimensions of access and exposure: eating practices of mothers living in the western Brazilian Amazon. *Physis* 31, 04. doi: 10.1590/S0103-73312021310404.

98. Scaciota LL, Jaime PC, Borges CA (2022) Development and validation of a guide to support public managers and retailers in promoting a healthy food environment. *J Public Health*. doi: 10.1007/s10389-021-01669-w.

99. Silva FMOD, Novaes TG, Ribeiro AQ et al. (2019) Environmental factors associated with obesity in the adult population in a medium-sized Brazilian city. *Cad Saude Publica* 35, 5, e00119618. doi: 10.1590/0102-311X00119618.

100. Silva TPR, Matozinhos FP, Gratão LHA et al. (2021a) Coexistence of risk factors for cardiovascular diseases among Brazilian adolescents: Individual characteristics and school environment. *PLoS One* 16, 7, e0254838. doi: 10.1371/journal.pone.0254838.

101. Silva TPRD, Viana TGF, Pessoa MC et al. (2022) Environmental and individual factors associated with gestational weight gain. *BMC Public Health* 22, 1, 540. doi: 10.1186/s12889-022-12948-w.

102. Silva ADDCE, Silva ARD, Hofelmann DA (2021b) Spatial distribution of public outlets for fruit and vegetable sales in Curitiba in the state of Paraná, Brazil. *Cien Saude Colet* 26, 8, 3111-3121. doi: 10.1590/1413-81232021268.04442020.

103. Soares ALG, França GVA, Gonçalves H (2014) Household food availability in Pelotas, Brazil: An approach to assess the obesogenic environme. *Rev Nutr* 27, 02. doi: 10.1590/1415-52732014000200006.

104. Souza LBO, Azevedo ABC, Bandoni DH et al. (2022) Characteristics of Brazilian school food and physical activity environments: PeNSE 2015. *Rev Saude Publica* 55, 115. doi: 10.11606/s1518-8787.2021055003377.

105. Tavares LF, Perez PMP, Dos Passos MEA et al. (2021) Development and Application of Healthiness Indicators for Commercial Establishments That Sell Foods for Immediate Consumption. *Foods* 10, 6, 1434. doi: 10.3390/foods10061434.

106. Vale D, Lyra CO, Santos TTD et al. (2021) Acceptance of school food by Brazilian adolescents: individual and school context determinants. *Cien Saude Colet* 26, 2, 637-650. doi: 10.1590/1413-81232021262.17392020.

107. Velásquez-Meléndez G, Mendes LL, Padez CM (2013) Built environment and social environment: associations with overweight and obesity in a sample of Brazilian adults. *Cad Saude Publica* 29, 10, 1988-96. doi: 10.1590/0102-311x00078112.

108. Vepsäläinen H, Mikkilä V, Erkkola M et al. (2015) Association between home and school food environments and dietary patterns among 9-11-year-old children in 12 countries. *Int J Obes Suppl* 5, 2, S66-73. doi: 10.1038/ijosup.2015.22.

109. Willets C, Santos IS, Lotufo PA et al. (2019) Association Between Perceived Neighborhood Characteristics and Carotid Artery Intima-Media Thickness: Cross-Sectional Results From the ELSA-Brasil Study. *Global Heart* 14, 4, 379–85. doi: 10.1016/j.gheart.2019.09.002.

110. Wognski ACP, Ponchek VL, Dibas EES et al. Commercialization of food in school canteens. *Braz J Food Technol* 22. doi: 10.1590/1981-6723.19818.

111. Zuccolotto DCC, Bertola MR, Isobe MT et al. (2013) Reproducibility of a questionnaire about perceived food environment and produce intake by pregnant women. *Rev Nutr* 26, 6, 727-735. doi: 10.1590/S1415-52732013000600011.

112. Zuccolotto DCC, Barbieri P, Sartorelli DS (2015) Food environment and family support in relation to fruit and vegetable intake in pregnant women. *ALAN* 65, 4, 216-224.

113. Glanz K, Sallis JF, Saelens BE et al. (2005) Healthy nutrition environments: concepts and measures. *Am J Health Promot* 19, 5, 330-3. doi: 10.4278/0890-1171-19.5.330.

114. Granheim SI (2019) The digital food environment. *UNSCN Nutrition* 44, 116–121.

115. Espinoza PG, Egaña D, Masferrer D et al. (2018) Propuesta de un modelo conceptual para el estudio de los ambientes alimentarios en Chile. *Rev Panam Salud Publica* 41, e169. doi: 10.26633/RPSP.2017.169.

116. Swinburn B, Sacks G, Vandevijvere S et al. (2013) INFORMAS (International Network for Food and Obesity/non-communicable diseases Research, Monitoring and Action Support): overview and key principles. *Obes Rev* 14, 1, 1-12.

117. Swinburn B, Egger G, Raza F (1999) Dissecting obesogenic environments: the development and application of a framework for identifying and prioritizing environmental interventions for obesity. *Prev Med* 29, 6, 563-570.

118. Story M, Kaphingst KM, Robinson-O’Brien R et al. (2008) Creating healthy food and eating environments: Policy and environmental approaches. *Annu Rev Public Health* 29, 1, 253-272. doi: 10.1146/annurev.publhealth.29.020907.090926.

119. Granheim SI, Opheim E, Terragni L et al. (2020) Mapping the digital food environment: a scoping review protocol. *BMJ Open* 10, 4, e036241. doi: 10.1136/bmjopen-2019-036241.

120. Food and Agriculture Organization of the United Nations (FAO) (2019) School food and nutrition framework. Rome: FAO.

121. Caspi CE, Sorensen G, Subramanian SV et al. (2012) The local food environment and diet: a systematic review. *Health Place* 18, 5, 1172-87. doi: 10.1016/j.healthplace.2012.05.006.

122. Monteiro LZ, de Farias JM, de Lima TR et al. (2022) Physical activity and perceived environment among adults from a city in Southern Brazilian. *Cien Saude Colet* 27, 6, 2197-210. doi: 10.1590/1413-81232022276.17372021.

123. Almeida IJ, Garcez A, Backes V et al. (2022) Association between the community food environment and dietary patterns in residents of areas of different socio-economic levels of a southern capital city in Brazil. *Br J Nutr* 1-9. doi: 10.1017/S0007114522001969.

124. Barbosa BB, Penha EDS, Carioca AAF. (2022) Food environment of the economic capital of the Northeast: social and territorial disparities in the availability of food stores. *Rev Nutr* 35, e210060. doi: 10.1590/1678-9865202235e210060.

125. Batista CHK, Leite FHM, Borges CA. (2022) Association between advertising patterns and ultra-processed food in small markets. *Cien Saude Colet* 27, 7, 2667-2678. doi: 10.1590/1413-81232022277.19122021.

126. Cordeiro NG, Mendes LL, Jardim MZ et al. (2022) Do Food and Nutrition Public Establishments Influence Availability to Healthy Food in Neighborhood? *J Hunger Environ Nutr*. doi: 10.1080/19320248.2022.2155095.

127. Silva FMO, Longo GZ, de Camargo AM et al. (2022) Neighborhood factors associated with leisure-time physical activity in a Brazilian city. *Health Promot Int* 37, 6, 1-12. doi: 10.1093/heapro/daac158.

128. Albuquerque FM, Pessoa MC, Filgueiras MDS et al. (2022) Neighborhood obesogenic environment and cardiometabolic risk in Brazilian children: The mediation role of the mother's body mass index. *Am J Hum Biol* e23835. doi: 10.1002/ajhb.23835.

129. Albuquerque FM, Pessoa MC, Filgueiras MDS et al. (2022) Obesogenic environment around schools is associated with atherogenic risk in Brazilian children. *Am J Hum Biol* 1-11. doi: 10.1002/ajhb.23844.

130. Assis MM, Gratão LHA, da Silva TPR et al. (2022) School environment and obesity in adolescents from a Brazilian metropolis: cross-sectional study. *BMC Public Health* 22, 1, 1229. doi: 10.1186/s12889-022-13592-0.

131. Menezes RCE, Oliveira JS, Almendra R et al. (2022) Influence of food environment on ultra-processed drinks consumption among an economically vulnerable population in a metropolitan area in Brazil: A multilevel analysis. *Health Place* 77, 102869. doi: 10.1016/j.healthplace.2022.102869.

132. Souza BB, Quialheiro A, Correa EM et al. (2022) Association between healthy food environment and metabolic syndrome, waist circumference, and systolic blood pressure in older adults in Southern Brazil. *Front Aging* 3, 922687. doi: 10.3389/fragi.2022.922687.

133. Franco AD, Canella DS, Tavares LF et al. (2022) Content validity and reliability of a university food environment assessment instrument. *Cien Saude Colet* 27, 6, 2385-2396. doi: 10.1590/1413-81232022276.13792021.

134. Grilo MF, Menezes C, Duran AC. (2022) Food swamps in Campinas, Brazil. *Cien Saude Colet* 27, 7, 2717-2728. doi: 10.1590/1413-81232022277.17772021.

135. Lopes MS, Martiniano MO, de Freitas PP et al. (2022) Sources of food ready to consume around the Health Academy Program units: an analysis according to inequality. *Cien Saude Colet* 27, 8, 3283-3294. doi: 10.1590/1413-81232022278.02232022.

136. Novaes TG, Mendes LL, Almeida LFF et al. (2022) Availability of food stores around Brazilian schools. *Cien Saude Colet* 27, 6, 2373-2383. doi: 10.1590/1413-81232022276.19372021.

137. Prado TR, Mazzonetto AC, Botelho AM, Fiates GMR. (2022) Home availability of ultraprocessed foods in families who prepare meals at home. *Rev Nutr* 35, e210249. doi: 10.1590/1678-9865202235e210249.

138. Serafim P, Borges CA, Cabral-Miranda W, Jaime PC. (2022) Ultra-Processed Food Availability and Sociodemographic Associated Factors in a Brazilian Municipality. *Front Nutr* 9, 858089. doi: 10.3389/fnut.2022.858089.

139. Oliveira JS, Menezes RCE, Almendra R et al. (2022) Unhealthy food environments that promote overweight and food insecurity in a brazilian metropolitan area: A case of a syndemic? *Food Policy* 112, 102375. doi: 10.1016/j.foodpol.2022.102375.
